# In Vitro Antigiardial Activity and In Silico Target Exploration of Quassinoids Isolated from *Castela tortuosa* Liebm.

**DOI:** 10.3390/ijms27198733

**Published:** 2026-09-30

**Authors:** Ulises Murrieta-Dionicio, Fernando Calzada, Elizabeth Barbosa, Miguel Valdes, Jesica Ramirez-Santos, Benito Reyes-Trejo, Holber Zuleta-Prada, Lino Reyes

**Affiliations:** 1 Laboratorio de Productos Naturales, Área de Química, Departamento de Preparatoria Agrícola, Universidad Autónoma Chapingo, Chapingo 56230, Mexico; murrieta.dionicio.091293@gmail.com (U.M.-D.); hzuletap@chapingo.mx (H.Z.-P.); 2 Unidad de Investigación Médica en Farmacología, UMAE Hospital de Especialidades, 2° Piso CORSE Centro Médico Nacional Siglo XXI, IMSS, Ciudad de Mexico 06720, Mexico; valdesguevaramiguel@gmail.com (M.V.); jes.ram.san@gmail.com (J.R.-S.); 3 Escuela Superior de Medicina, Instituto Politécnico Nacional, Ciudad de Mexico 07738, Mexico; rebc78@yahoo.com.mx; 4 Laboratorio de Inmunología, Departamento de Sistemas Biológicos, Universidad Autónoma Metropolitana Unidad Xochimilco, Calz. del Hueso 1100, Coapa, Villa Quietud, Coyoacán, Ciudad de Mexico 04960, Mexico; 5 Departamento de Química Orgánica, Facultad de Química, Universidad Nacional Autónoma de México, Ciudad Universitaria, Delegación Coyoacán 04510, Mexico; linoj23@quimica.unam.mx

**Keywords:** *Castela tortuosa*, quassinoids, chaparrinone, *Giardia lamblia*, antigiardial activity, in vitro and in silico studies

## Abstract

Giardiasis is a parasitic disease for which there is a growing need to develop new therapeutic alternatives due to the adverse effects associated with first-line treatments such as metronidazole and the increasing emergence of resistant strains. In this study, the in vitro antigiardial activity of three quassinoids, chaparrin (**1**), amarolide (**2**), and chaparrinone (**3**), isolated from the stems of *Castela tortuosa* Liebm., was evaluated. The compounds were obtained from the methanolic extract through fractionation and chromatographic purification, and their antigiardial activity was determined against *Giardia lamblia* trophozoites. In addition, in silico studies were conducted to evaluate their physicochemical, pharmacokinetic, and toxicological properties, as well as molecular docking studies against actin, aldose reductase (ARL), and pyruvate: ferredoxin oxidoreductase (PFOR). Chaparrinone (**3**) exhibited the highest antigiardial activity, with an IC_50_ 18.98 μg mL^−1^, whereas chaparrin (**1**) and amarolide (**3**) showed lower activity. Amarolide (**2**) exhibited the highest binding affinities toward the molecular targets, while chaparrin (**1**) showed the most favorable overall toxicological/ADME profile. Taken together, these findings highlight chaparrinone (**3**) as a quassinoid of interest for the development of new antigiardial agents and support the need for further enzymatic, pharmacokinetic, and in vivo studies to more precisely determine its therapeutic potential.

## 1. Introduction

Giardiasis is a parasitic disease caused by the flagellated protozoan *Giardia lamblia* (syn. *Giardia duodenalis* or *Giardia intestinalis*). Infection is acquired through the ingestion of water or food contaminated with fecal matter [1]. Giardiasis affects approximately 200–300 million people annually, making *G. lamblia* one of the major causative agents of diarrheal disease worldwide, particularly in low-income and developing countries [2,3]. The clinical manifestations of giardiasis encompass a broad spectrum, ranging from asymptomatic infections to abdominal pain, chronic diarrhea, nausea, and vomiting, as well as weight loss, and impaired nutrient absorption [4].

Pharmacological treatment of giardiasis has relied primarily on nitroimidazole derivatives, with metronidazole being considered the first-line treatment in most countries [5]. However, metronidazole may cause several adverse effects, including headache, metallic taste, dark urine, and dizziness, as well as nausea, and hypersensitivity reactions [4,6]. In addition to these limitations, the emergence of increasingly frequent drug-resistant strains has become a significant concern [7]. Second-line drugs, including albendazole, nitazoxanide, furazolidone, and paromomycin, have consequently been developed and implemented. Nevertheless, these agents may exhibit lower efficacy and cause adverse effects similar to those associated with metronidazole [8].

In this context, the search for new therapeutic alternatives has stimulated the investigation of plant-derived antigiardial agents as promising complementary treatments, particularly because of their potentially low toxicity [9]. Among these natural products are quassinoids, a group of highly oxygenated diterpenoids characteristic of the tree and shrub species belonging to the Simaroubaceae family. Based on the number of carbon atoms in their carbon skeleton, quassinoids are classified into five main groups: C_18_, C_19_, C_20_, C_22_, and C_25_ [10,11]. Several quassinoids isolated from the fruits of Simaroubaceae species, including *Brucea javanica* and *Simarouba amara*, have exhibited selective antiprotozoal activity against medically important protozoa, including *Plasmodium falciparum*, *Giardia lamblia*, and *Toxoplasma gondii* [12].

*Castela tortuosa* Liebm. (Simaroubaceae), commonly known as “chaparro amargoso”, is a woody shrub approximately 1–2.5 m in height, distributed from southwestern Texas to northeastern Mexico. In Mexico, this species has traditionally been used to treat gastrointestinal infections and spasmodic pain, mainly in the form of infusions [13]. Previous phytochemical studies have demonstrated that *C. tortuosa* Liebm. is a source of bioactive quassinoids, some of which have exhibited selective antiprotozoal activities, particularly against *Plasmodium falciparum* [14].

Therefore, with the aim of contributing to the identification of new natural alternatives for the treatment of giardiasis, and considering both the antiparasitic potential of quassinoids and the phytochemical and ethnomedicinal background of *C. tortuosa* Liebm., the present study aimed to evaluate the antigiardial activity by in vitro and in silico approaches of chaparrin, amarolide, and chaparrinone (compounds **1**–**3**, Figure 1), three quassinoids isolated from *C. tortuosa* Liebm., against *G. lamblia* trophozoites. The findings provide further evidence supporting the potential of medicinal plants and their bioactive constituents as sources of novel therapeutic agents against *Giardia* infections.

## 2. Results

### 2.1. Quassinoids from C. tortuosa Liebm.

Three quassinoids were isolated from the stems of *C. tortuosa* Liebm., as described in the Materials and Methods section. Their structures were established by analysis of their IR, ^1^H and ^13^C NMR, HSQC, COSY and HMBC spectroscopic data and confirmed by comparison with the corresponding data reported in the literature [14,15,16] (Figures S1–S28).

**Chaparrin** (**1**): White amorphous powder. IR (ATR, $\nu_{\max}$, cm^−1^): 3466, 3385, 3189, 2972, 2867, 1725, 1516, 1387, 1213, 1173, 1038, 1017, 964, 920, 838, 792, and 722. The ^1^H and ^13^C NMR spectroscopic data are provided in Table S1.

**Amarolide** (**2**): Colorless crystals. IR (ATR, $\nu_{\max}$, cm^−1^): 3507, 3478, 2960, 2919, 2863, 1723, 1703, 1458, 1424, 1388, 1352, 1263, 1232, 1121, 1079, 1039, 972, 937, 883, 822, and 706. The ^1^H and ^13^C NMR spectroscopic data are provided in Table S2.

**Chaparrinone** (**3**): White amorphous powder. IR (ATR, $\nu_{\max}$, cm^−1^): 3464, 3422, 3166, 2932, 2890, 1725, 1664, 1621, 1386, 1363, 1229, 1182, 1123, 1077, 1042, 1024, 961, 916, 878, 854, 797, and 699. The ^1^H and ^13^C NMR spectroscopic data are provided in Table S3.

### 2.2. Antigiardial Activity

Fractionation of the methanolic extract of *C. tortuosa* Liebm. resulted in the isolation of three quassinoids with giardicidal activity. The antigiardial activity of the tested compounds is summarized in Table 1. Among these quassinoids, chaparrinone was the most potent compound with an IC_50_ value of 18.98 μg mL^−1^. It was less active than metronidazole, the antigiardial drug used as the control; however, it showed an effect similar to that of kaempferol and tiliroside, flavonoids reported to have significant in vitro and in vivo antigiardial activity [17,18]. Chaparrin and amarolide showed weak antigiardial activity.

### 2.3. In Silico Physicochemical, Pharmacokinetic and Toxicological Profile of Chaparrin, Amarolide and Chaparrinone

The physicochemical, pharmacokinetic and toxicological properties of chaparrin, amarolide and chaparrinone were predicted using different in silico platforms and compared with metronidazole as the reference compound (Table S4).

Regarding physicochemical properties, the three quassinoids presented MW ranging from 364.44 to 380.44 g mol^−1^, remaining below the threshold of 500 g mol^−1^ established for orally active compounds. Similarly, all quassinoids had moderate lipophilicity (LogP = 1.36–1.77), comparable to that of metronidazole (LogP = −0.102), although quassinoids were slightly more lipophilic. Water solubility (LogS) predictions indicated that chaparrinone (−3.957) and chaparrin (−3.396) were less soluble than amarolide (−2.133) and metronidazole (−0.208), suggesting reduced aqueous solubility for these quassinoids. Topological polar surface area (TPSA) values ranged from 100.90 to 116.45 Å^2^, remaining below the threshold of 140 Å^2^ generally associated with favorable intestinal permeability. In addition, none of the three compounds has any rotatable bonds and they exhibit an adequate balance between hydrogen bond donors (2–4) and acceptors (6–7), indicating conformational stiffness while maintaining an acceptable hydrogen bonding capacity.

Drug-likeness analysis demonstrated that chaparrin, amarolide and chaparrinone fulfilled all four major drug-likeness filters (Lipinski, Ghose, Veber and Egan), similarly to metronidazole, supporting their potential suitability as orally bioavailable drug candidates.

The predicted absorption profile showed that the three quassinoids exhibited good human intestinal absorption, with HIA values ranging from 67.73 to 70.89%, although slightly lower than metronidazole (80.28%). Caco-2 permeability values were similar among the quassinoids (−5.448 to −5.730) and lower than metronidazole (−4.712), indicating comparatively reduced passive intestinal permeability. Distribution predictions indicated moderate plasma protein binding for the quassinoids (34.83–42.81%), which was approximately two-fold higher than that predicted for metronidazole (17.40%). Blood–brain barrier penetration probabilities ranged from 15.07 to 25.74%, values that were slightly lower than those predicted for metronidazole (32.36%), suggesting a limited exposure of the central nervous system. The predicted distribution volumes varied between the compounds, with chaparrinone showing the highest value (1.230 L kg^−1^), followed by metronidazole (0.830 L kg^−1^), chaparrin (0.657 L kg^−1^) and amarolide (0.515 L kg^−1^).

The metabolic profile obtained revealed a low risk of cytochrome P450-mediated drug interactions. According to the predictions obtained, none of the compounds inhibit the isoenzymes CYP1A2, CYP2C19, CYP2C9, CYP2D6 or CYP3A4. Similarly, none were identified as CYP2D6 substrates. However, it was predicted that amarolide, chaparrinone and metronidazole would act as CYP2C19 substrates, while chaparrin was not.

Regarding excretion parameters, amarolide showed the highest elimination (13.081), followed by metronidazole (6.299), chaparrinone (2.665) and chaparrin (2.607). The predicted elimination half-life was relatively similar among quassinoids (0.796–0.826 h) and slightly longer than that of metronidazole (0.525 h), suggesting comparable elimination kinetics.

The toxicity profile revealed a favorable overall safety profile for chaparrin and chaparrinone. Both compounds were predicted to be non-mutagenic, non-carcinogenic, non-neurotoxic, non-nephrotoxic, and non-cardiotoxic. In contrast, amarolide was predicted to exhibit mutagenic activity, although it remained negative for carcinogenicity and organ-specific toxicities. Metronidazole was predicted to possess mutagenic, carcinogenic, and nephrotoxic potential, consistent with its known toxicological profile. Prediction of human hepatotoxicity indicated a low to moderate probability of chaparrin (+) and high probability of metronidazole (+++), while amarolide and chaparrinone were predicted to lack hepatotoxic potential. Predictions of acute oral toxicity classified chaparrin and metronidazole as class IV (harmful if ingested), amarolide as class V (may be harmful if ingested), and chaparrinone as class II (toxic if ingested), indicating that chaparrinone showed the lowest predicted LD_50_ among the quassinoids evaluated, despite its favorable toxicity profile.

Overall, these in silico analyses suggest that chaparrin, amarolide, and chaparrinone possess physicochemical characteristics consistent with oral drug development, meet the primary criteria for drug-likeness, and exhibit low cytochrome P450 inhibition potential. Among the three quassinoids, chaparrin showed the most balanced pharmacokinetic and toxicological profile, while chaparrinone combined favorable ADME characteristics with unfavorable acute oral toxicity, and amarolide showed the highest clearance but predicted mutagenic potential. Compared with metronidazole, quassinoids generally showed lower intestinal permeability but a more favorable overall toxicity profile, particularly in carcinogenicity, nephrotoxicity, and hepatotoxicity.

### 2.4. Molecular Docking Study

To elucidate the potential antigiardial mechanism of action of the quassinoids evaluated in this study (amarolide, chaparrin, and chaparrinone), in silico molecular docking studies focused on three protein targets of high physiological relevance for *Giardia lamblia*: actin, pyruvate: ferredoxin oxidoreductase (PFOR), and aldose reductase (ALR). The molecular docking studies revealed the key interactions of chaparrin, amarolide, and chaparrinone against the target proteins. The local docking analysis allowed for the identification of the binding affinities and binding positions of the quassinoids within the target structures.

#### 2.4.1. Molecular Docking Analysis of Quassinoids on Actin

Docking analysis showed that the reference drug metronidazole exhibited a binding energy (∆G) of −6.53 kcal mol^−1^ and an estimated inhibition constant (Ki) of 25.32 μM. Its binding profile involved polar interactions with Glu335 and Tyr338, a non-polar interaction with Lys337, and an additional contact with Pro334 (Figure 2D). Among the evaluated quassinoids, amarolide demonstrated the highest binding affinity for actin, significantly outperforming the reference drug. Amarolide yielded a ∆G of −8.64 kcal mol^−1^ and Ki of 467.96 nM. This pronounced affinity is driven by an extensive network of polar interactions with Met111, Ile176, Leu177, Arg178, Leu179, Asp188, Met191, Thr195, Thr202, and Arg207, alongside a non-polar interaction with Lys192 (Figure 2A). Chaparrinone and chaparrin also exhibited favorable binding energies, ∆G of −6.66 kcal mol^−1^ (Ki = 13.14 μM) and −6.47 kcal mol^−1^ (Ki = 18.15 μM), respectively. Chaparrinone formed polar interactions with Met111, His174, Ile176, Leu177, Arg178, Leu179, Asp188, Thr195, Phe201, Thr202, and Arg209, coupled with non-polar interactions with Met191 and Lys192 (Figure 2C). Similarly, chaparrin interacted via polar bonds with His174, Ile176, Leu177, Arg178, Asp188, Tyr189, Met191, Phe201, Thr202 and Arg207, displaying non-polar interactions with Met111, Pro173 and Lys192 (Figure 2B).

It is noteworthy that all three quassinoids share a common binding pocket on the actin molecule, as evidenced by conserved interactions with key amino acid residues Ile176, Leu177, Arg178, Asp188, Lys192, and Thr202. This distinct binding behavior, compared to the metronidazole interaction site, suggests an allosteric interaction over the actin structure that could be responsible for the disruption of the Giardia cytoskeleton.

#### 2.4.2. Molecular Docking Analysis of Quassinoids on Aldose Reductase (ALR)

For the enzyme, fidarestat was utilized as the reference ligand, yielding a ∆G of −6.52 kcal mol^−1^ and an estimated Ki of 15.52 μM. The binding stabilization of fidarestat was primarily driven by polar interactions with Val39, Tyr40, Trp73, Phe118, Tyr210, Cys301, Phe306, and Trp307, supplemented by a non-polar interaction with Trp12 (Figure 3D). Consistent with the trend observed in the actin model, all three quassinoids exhibited superior thermodynamic affinities for ALR compared to the reference. Amarolide displayed the most potent binding profile, achieving a ∆G of −10.48 kcal mol^−1^ and Ki of 20.85 nM. This highly stable complex was formed through polar interactions with Val39, Trp73, Trp105, Cys301, and Phe306, alongside a strong network of non-polar interactions involving Trp12, Tyr40, His104, Phe118, and Trp307 (Figure 3A). Chaparrin also demonstrated a remarkably high affinity for ALR, with a ∆G of −9.22 kcal mol^−1^ (Ki = 173.19 nM). Its interaction profile included polar bonds with Gln13, Val39, Tyr40, Trp73, His104, Trp105, Tyr210, Gln220, and Trp307, complemented by non-polar interactions with Trp12, Phe118, Cys301, and Phe306 (Figure 3B). Similarly, chaparrinone surpassed the reference inhibitor, presenting a binding energy of −7.80 kcal mol^−1^ (Ki = 1.92 μM). The stabilization of chaparrinone within the ALR active site was mediated by polar interactions with Gln13, Trp105, Ser209, Gln220, Phe300, Cys301, Asp302, and Ile304, as well as non-polar interactions with Trp12, Tyr210, and Phe306 (Figure 3C).

Docking results indicate that amarolide, chaparrin, and chaparrinone are capable of effectively binding to the ALR enzyme. The conservation of specific anchoring residues across the evaluated ligands, particularly the non-polar interaction with Trp12, suggests a localized consensus in their mechanism of target recognition. These findings support the hypothesis that ALR constitutes a viable pharmacological target for the antigiardial activity exerted by these quassinoids.

#### 2.4.3. Molecular Docking Analysis of Quassinoids on Pyruvate: Ferredoxin Oxidoreductase (PFOR)

To evaluate the interactions within the PFOR enzyme, metronidazole was employed as the reference drug. It presented a binding energy ∆G of −3.67 kcal mol^−1^ and Ki of 2.05 mM. Its binding profile within the PFOR active site was characterized by polar interactions with Asp9, Phe174, Glu179, Ile180, Lys435, Tyr452, and Phe453, alongside a single non-polar interaction with Tyr455 (Figure 4D).

In stark contrast to the reference drug, all evaluated quassinoids exhibited significantly higher thermodynamic affinities for the PFOR enzyme. Amarolide displayed the most robust binding profile, achieving a ∆G of −8.87 kcal mol^−1^ and Ki of 314.75 nM. The structural stabilization of amarolide was driven by an extensive network of polar interactions involving Met5, Ser145, Gln147, Glu148, Gln181, Ile183, Glu184, Pro474, Ile475 and Gln476, complemented by a non-polar interaction with Val146 (Figure 4A). Chaparrin also demonstrated a remarkably strong affinity for PFOR, yielding a binding energy of −8.76 kcal mol^−1^ (Ki = 382.07 nM). This complex was primarily stabilized through multiple polar bonds with Gly424, Gly426, Ala427, Asp428, Gly429, Thr430, Gly432, Asp456, Lys459, Ser460, Gly461, Gly462, Ile463, and Thr464, as well as non-polar interaction with Val431 and Tyr455 (Figure 4B). Finally, chaparrinone surpassed the reference drug with a ∆G of −7.78 kcal mol^−1^ (Ki = 1.97 μM). The interaction profile for chaparrinone included polar bonds with Ser145, Val146, Gln147, Glu148, Gln181, Ile183, Glu281, Glu284, Lys301, Arg303, Phe448, Pro474, Ile475, and Gln476. Additionally, it formed non-polar interactions with Arg256 and Tyr258 (Figure 4C).

Overall, the molecular docking study results for PFOR highlight that amarolide, chaparrin, and chaparrinone possess a markedly superior binding affinity compared to metronidazole. The diverse interaction networks, particularly the strong nanomolar inhibition constants predicted for amarolide and chaparrin, strongly support the potential of these quassinoids to effectively target and inhibit PFOR, corroborating their antigiardial activity.

## 3. Discussion

Giardiasis remains an important intestinal parasitic disease worldwide and represents a significant cause of acute and persistent diarrheal disease, particularly in vulnerable populations and settings with inadequate sanitation. Although several drugs, including nitroimidazoles, benzimidazoles, nitazoxanide, and paromomycin, are available for treatment, therapeutic failure and reduced susceptibility to commonly used agents, particularly metronidazole, have increasingly been reported, highlighting the need for new therapeutic alternatives with distinct pharmacological profiles [19]. In this context, medicinal plants represent a valuable source of structurally diverse bioactive metabolites with potential antigiardial activity. A systematic review identified 57 plant species from 19 botanical families with experimental evidence of activity against *Giardia*, including studies performed in vitro and in vivo, supporting the potential of plant-derived products as a source of alternative antigiardial agents [20]. The present study aimed to evaluate the in vitro antigiardial activity of chaparrin, amarolide and chaparrinone, three quassinoids isolated from *C. tortuosa* Liebm., against trophozoites of *G. lamblia*, and to characterize their pharmacological potential by in silico analysis of their ADME and toxicological properties, as well as to explore their possible interactions with relevant molecular targets by molecular docking.

When evaluating the purified quassinoids, chaparrinone emerged as the most active compound, exhibiting an IC_50_ of 18.98 µg mL^−1^, followed by amarolide (126.04 µg mL^−1^) and chaparrin (283.80 µg mL^−1^). This biological profile is consistent with previous research highlighting quassinoids, commonly isolated from *Castela* species, as metabolites with broad-spectrum antiparasitic efficacy [21,22]. In particular, chaparrinone has previously exhibited potent antiplasmodial activity against *Plasmodium falciparum*, with an IC_50_ of 0.037 µg mL^−1^, while chaparrin isolated from *Castela texana* produced complete inhibition of *Entamoeba histolytica* growth at 100 µg mL^−1^ [23]. The differential activity among the structurally related quassinoids suggests that subtle changes in oxygenation and functional-group distribution may influence giardicidal potency. In the present study, antigiardial activity increased markedly following fractionation, with the IC_50_ decreasing from 507.60 µg mL^−1^ for the methanolic extract to 67.42 µg mL^−1^ for the dichloromethane fraction and to 18.98 µg mL^−1^ for chaparrinone. This progressive increase in potency suggests that fractionation enriched the antigiardial constituents of *C. tortuosa*, with chaparrinone emerging as the most active compound among those evaluated.

The in silico ADME predictions showed that chaparrin, amarolide and chaparrinone had physicochemical characteristics compatible with orally active drug candidates and were more favorable than those of metronidazole in several pharmacokinetic parameters. The three quassinoids comply all four major drug-likeness rules including Lipinski, Ghose, Veber, and Egan, indicating that their molecular weight, lipophilicity, polarity, and hydrogen bonding capacity are within the ranges generally associated with optimal oral bioavailability. These findings are consistent with the structural characteristics of quassinoids, which are highly oxygenated triterpenoid derivatives that generally possess moderate lipophilicity and conformational stiffness. These structural features have been associated with well-defined structure–activity relationships and have attracted considerable interest in drug discovery despite their relatively complex molecular architecture [24]. Although its lower aqueous solubility and lower permeability to Caco-2 compared to metronidazole suggest limited passive membrane diffusion, intestinal absorption of natural products is typically influenced by intestinal uptake and metabolism transporters, allowing several phytochemicals to show optimal or moderate oral bioavailability despite relatively low in vitro permeability [25,26]. Similar pharmacokinetic behavior has been described for quassinoids, whose highly oxygenated and structurally complex scaffolds are often associated with limited oral bioavailability, highlighting the need for further experimental pharmacokinetic evaluation [27]. In addition, prediction of moderate plasma protein binding and low penetration of the blood–brain barrier suggests a favorable distribution profile, which could provide adequate systemic exposure and minimize undesirable central nervous system effects. Additionally, bioinformatic predictions showed that none of the compounds are inhibitors of any of the main CYP450 isoforms, suggesting a low risk of clinically relevant drug–drug interactions. Although amarolide and chaparrinone were predicted to be CYP2C19 substrates, substrate recognition does not necessarily represent a pharmacokinetic limitation but rather indicates their possible metabolic pathway. Overall, these findings suggest that the quassinoids evaluated may show a favorable pharmacokinetic profile for further drug development [28].

From a toxicological point of view, chaparrin showed the most favorable safety profile, being predicted as non-mutagenic, non-carcinogenic, non-neurotoxic, non-nephrotoxic and non-cardiotoxic. Chaparrinone showed a similar toxicity profile but was classified as acute oral toxicity class II, indicating a higher expected acute toxicity, while amarolide was shown to be a mutagenic agent. In contrast, metronidazole showed mutagenicity, carcinogenicity, nephrotoxicity, and an increased likelihood of hepatotoxicity, which is consistent with previous experimental studies that demonstrated mutagenic and carcinogenic effects following prolonged exposure in animal models, although evidence of hepatotoxicity and nephrotoxicity in humans remains limited [29]. Overall, these findings suggest that chaparrin represents the most promising candidate among the quassinoids evaluated due to its balanced ADME/Tox profile. However, experimental pharmacokinetic studies, including intestinal permeability, metabolic stability, plasma protein binding and in vivo toxicity assays, are required to validate these computational predictions.

To explore potential molecular targets underlying the observed antigiardial activity, in silico molecular docking analyses were performed against three protein targets of high physiological relevance for *Giardia lamblia*: actin, pyruvate: ferredoxin oxidoreductase (PFOR), and aldose reductase (ALR) [30]. Interestingly, the molecular docking studies revealed that amarolide is the ligand with the highest thermodynamic affinity for the three evaluated enzymes. In the metabolic enzyme PFOR, amarolide formed an extensive network of polar interactions, stabilizing a complex with a binding energy (∆G) of −8.87 kcal mol^−1^, a value superior to that of metronidazole (∆G = −3.67 kcal mol^−1^). Similarly, in the ALR model, amarolide formed the most stable complex with a ∆G of −10.48 kcal mol^−1^, and in actin, it presented a ∆G of −8.64 kcal mol^−1^. This distinct binding behavior suggests an allosteric interaction over the actin structure that could be responsible for the disruption of the parasite’s cytoskeleton [31].

Of pharmacological interest is the discrepancy observed between the theoretical enzymatic affinity and the integral cellular activity: while amarolide demonstrated the most potent binding profile in silico, chaparrinone was nearly 6.6 times more potent in the in vitro biological assay with intact trophozoites. This phenomenon demonstrates that giardicidal efficacy does not depend exclusively on the binding strength to the enzymatic target. The overwhelming superiority of chaparrinone in vitro can be explained by key differences in its physicochemical properties, suggesting an optimal partition coefficient that grants it greater permeability to cross the parasite’s biological membrane and reach the intracellular space more easily than amarolide [32]. Additionally, the structural profile of chaparrinone could potentially involve multiple molecular targets, or generate an endogenous oxidative stress that proves lethal and acts in parallel or independently of the exclusive inhibition of PFOR or ALR [33,34].

Taken together, our findings suggest that the evaluated quassinoids exhibit a favorable interaction with proteins relevant to *Giardia lamblia* survival, particularly PFOR and ALR. However, despite the promising in vitro antigiardial activity, this study has certain limitations that must be addressed in future research. First, while the molecular docking approaches indicate high binding affinities, they are purely exploratory; the lack of molecular dynamics (MD) simulations prevents the assessment of the long-term thermodynamic stability of the predicted protein-ligand complexes. Second, the current limited annotation of functional networks and interactomes for *G. lamblia* restricts the feasibility of a robust network pharmacology analysis, which could otherwise elucidate broader multi-target mechanisms. Therefore, the docking results should be considered as a hypothesis about potential molecular targets and not as definitive evidence of inhibition. Future work will focus on validating these molecular targets through specific purified enzymatic assays, assessing the stability of the complexes via MD simulations, and determining the in vivo pharmacokinetic profile, cytotoxicity, and efficacy of chaparrinone as a lead compound in murine models of giardiasis.

## 4. Materials and Methods

### 4.1. General Experimental Procedures

Thin-layer chromatography (TLC) was performed on silica gel 60 F_254_ plates (Merck, Darmstadt, Germany). TLC plates were visualized under UV light (254 and 365 nm) and by spraying with a cobalt (II) chloride (CoCl_2_) reagent, followed by heating at 110 °C until color development. Column chromatography was carried out on silica gel 60 (0.063–0.200 mm; Macherey–Nagel, Düren, Germany). Infrared (IR) spectra were recorded using a Cary 630 FTIR spectrometer (Agilent Technologies, Santa Clara, CA, USA). ^1^H and ^13^C NMR spectra were recorded at 400 MHz and 100 MHz, respectively, using an Agilent DD2 400 MHz spectrometer (Agilent Technologies, Santa Clara, CA, USA). All NMR experiments were performed at 298 K in deuterated solvents. Approximately 10 mg of each sample was dissolved in DMSO-*d*_6_, pyridine-*d*_5_ or CDCl_3_. Chemical shifts (δ) were referenced to tetramethylsilane (TMS, *δ* = 0.00 ppm) as the internal standard and are reported in parts per million (ppm). Coupling constants (*J*) are reported in Hertz (Hz). TMS, DMSO-*d*_6_, pyridine-*d*_5_, CDCl_3_ and CoCl_2_ were purchased from Sigma-Aldrich (St. Louis, MO, USA). Analytical-grade solvents used for extraction and chromatographic separations were purchased from Merck (Darmstadt, Germany).

### 4.2. Plant Material

The *C. tortuosa* Liebm. plant material used in this study was collected in December 2025 in Tehuitzingo, Puebla, Mexico (18°19′09″ N, 98°16′38″ W). The species was identified by M.C. Antonio Cortés J. from the Universidad Autónoma Chapingo (UACh), and a voucher specimen (No. 37024) was deposited in the “Jorge Espinosa Salas” Herbarium-Hortorium at UACh.

### 4.3. Extraction and Purification of Quassinoids from C. tortuosa Liebm.

Dried stems of *C. tortuosa* Liebm. (2.5 kg) were extracted with methanol (3 × 5 L, 3 h each) at 60 °C. The combined methanolic extracts were concentrated under reduced pressure to afford a dark green crude extract (72.7 g). The plant material was extracted with methanol to obtain low-, medium-, and high-polarity secondary metabolites. The methanolic extract was subsequently subjected to successive liquid–liquid partitioning with *n*–hexane, CH_2_Cl_2_, EtOAc, and *n*–BuOH, yielding the corresponding fractions: *n*–hexane (6.72 g), CH_2_Cl_2_ (1.30 g), EtOAc (1.70 g), and *n*–BuOH (4.18 g). During partitioning with *n*–hexane, an intermediate layer consisting of a brown granular solid was formed. This insoluble material was collected by vacuum filtration, washed successively with CH_2_Cl_2_ and methanol, and dried to afford compound **1** as an amorphous white powder (807.2 mg). The CH_2_Cl_2_ fraction was dissolved in acetone producing a white precipitate that was recovered by vacuum filtration and washed with acetone. The resulting solid was recrystallized from CH_2_Cl_2_–acetone (1:1, *v*/*v*) to afford colorless crystals of compound **2** (98.1 mg). It is important to highlight that in this research only the CH_2_Cl_2_ was evaluated and purified considering that antiamoebic activity has been previously reported [21]. The acetone-soluble portion of the CH_2_Cl_2_ fraction (1.20 g) was adsorbed onto silica gel (2.5 g, Silica gel 60, 0.063–0.200 mesh, Macherey-Nagel, Düren, Germany); after that, it was subjected to silica gel column chromatography (C1; 50 g, Silica gel 60, 0.063–0.200 mesh, Macherey-Nagel, Düren, Germany; 20 cm in length and 2.7 cm in internal diameter) using a CH_2_Cl_2_-MeOH gradient [100:0 (66 fractions), 98:2 (30 fractions), 96:4 (15 fractions), 90:10 (30 fractions), 80:20 (15 fractions), and 0:100 (nine fractions), *v*/*v*] as the mobile phase. A total of 165 fractions were collected of 30 mL each, monitored by TLC, and combined according to their chromatographic profiles. Fractions C1-F69-79 yielded a white precipitate, which was washed with CH_2_Cl_2_ and collected by vacuum filtration to afford compound **3** as an amorphous white powder (17.1 mg).

### 4.4. Antigiardial Tests

*Giardia lamblia* strain WB, used in all experiments, was grown at 37 °C in TYI-S-33 modified medium supplemented with 10% calf serum, bovine bile, and penicillin–streptomycin (0.1%, Gibco, Grand Island, NY, USA). When the logarithmic phase of growth was reached, the trophozoites were detached from the medium and used. Antibiotics were omitted during experimental assays. In vitro susceptibility tests were performed using *G. lamblia* (5 × 10^4^) trophozoites that were incubated for 48 h at 37 °C in the presence of different concentrations (1.25–200 µg mL^−1^) of the crude extract, fraction, or pure compounds in DMSO (Sigma-Aldrich, St. Louis, MO, USA) at 2%. Each test included metronidazole as a standard giardicidal drug, a control (culture medium plus trophozoites and DMSO), and a blank (culture medium). After incubation, the trophozoites were detached by chilling, and 50 µL samples of each tube were subcultured in a fresh medium for another 48 h. The final number of parasites was determined with a hemocytometer. Then, data were analyzed using probit analysis. The percentage of trophozoites surviving was calculated by comparison with the growth in the control group. The plot of probit against log concentration was made, the best straight line was determined by regression analysis, and the IC_50_ values were calculated. The regression coefficient, its level of significance (*p* < 0.05 indicates a significant difference between groups), and the correlation coefficient were calculated, and 95% confidence interval (CI) values were determined. Results were analyzed using GraphPad Prism version 5.0 (San Diego, CA, USA) software. One-way ANOVA followed by Bonferroni’s multiple-comparison test was performed.

### 4.5. In Silico Physicochemical, Pharmacokinetic and Toxicological Profile

The physicochemical, pharmacokinetic, and toxicological properties of chaparrin, amarolide, chaparrinone, and metronidazole were predicted using the SwissADME [35], ADMETlab 3.0 [36], AI-DrugLab [37], and Tox-Prediction [38] web servers. The predicted parameters included physicochemical descriptors, drug-likeness, absorption, distribution, metabolism, excretion, cytochrome P450 interactions, and toxicity.

### 4.6. In Silico Studies

#### 4.6.1. Macromolecular Target Preparation and Optimization

The 3D crystallographic coordinates of the proteins were retrieved from the PDB and UniProt: actin (B1N2P0), aldose reductase (3KRB), and pyruvate: ferredoxin oxidoreductase (1KEK). Structures were optimized by removing water molecules, co-solvents, and non-catalytic heteroatoms, while preserving essential ions at the catalytic core. Polar hydrogens were added, protonation states were assigned at physiological pH (7.4), and Gasteiger charges were computed to generate topology files.

#### 4.6.2. Ligand Generation and Energetic Minimization

The 2D structures of metronidazole (CID: 445070) and fidarestat (CID: 160024) were retrieved from PubChem https://pubchem.ncbi.nlm.nih.gov/ (accessed on 1 June 2026). The structures of amarolide, chaparrin, and chaparrinone were drawn using ChemDraw Professional version 22.0.0.22 (PerkinElmer, Waltham, MA, USA) and subsequently optimized through energy and geometry minimization using Avogadro software (v 1.2.0). Optimization was driven by the UFF force field, combining a steepest-descent algorithm [39].

### 4.7. Molecular Docking Simulations

The molecular docking simulations to determine binding affinity (ΔG, kcal mol^−1^) were performed using AutoDock 4.2 [40]. A blind docking approach was intentionally implemented to ensure an unbiased exploration of the entire protein surface, allowing the identification of potential allosteric binding sites. This strategy was selected due to the highly oxygenated and rigid structural scaffold of quassinoids differs significantly from conventional co-crystallized inhibitors. The blind docking approach was implemented employing a 126 × 126 × 126 Å grid box (0.375 Å spacing) positioned at the geometric center of each protein to cover its entire surface. Conformational sampling utilized the Lamarckian genetic algorithm (LGA) with the following parameters: an initial population of 100 individuals, 1.0 × 10^7^ energy evaluations, and 27,000 generations. To validate the docking protocol, the respective co-crystallized ligands were re-docked into their target active sites; the obtained RMSD values were 1.9 for actin, 1.8 for aldose reductase, and 1.99 for pyruvate: ferredoxin oxidoreductase. One hundred independent runs were performed per system, and the resulting conformations were clustered. The lowest energy poses from the most populated clusters were selected to analyze 2D and 3D intermolecular interaction networks using BIOVIA Discovery Studio Visualizer (V 20.2.0.19295) [41], and PYMOL Molecular Graphics System (V3.0, Schrödinger, LLC), respectively.

## 5. Conclusions

The results obtained support the ethnomedicinal use of *C. tortuosa* Liebm. for the treatment of parasitic diseases and suggest its potential as a source of bioactive compounds with antigiardial activity. Extraction and fractionation of the methanolic extract obtained from the stems of *C. tortuosa* Liebm. enabled the isolation and identification of three quassinoids, chaparrin (**1**), amarolide (**2**), and chaparrinone (**3**), whose antigiardial activity was evaluated through in vitro assays. Among the compounds evaluated, chaparrinone (**3**) exhibited the highest activity against *G. lamblia* trophozoites, with an IC_50_ of 18.98 μg mL^−1^, being considerably more active than chaparrin (**1**) and amarolide (**2**). This result positions chaparrinone (**3**) as the most promising compound of this series for further studies, despite being obtained in lower yield compared with chaparrin (**1**), which was identified as the major quassinoid in *C. tortuosa* Liebm.

On the other hand, the in silico studies showed that the three quassinoids exhibit favorable physicochemical properties and drug-likeness characteristics associated with the development of orally administered drugs. Regarding their toxicological/ADME profiles, chaparrin (**1**) exhibited the most favorable overall profile, whereas amarolide (**2**) showed higher binding affinity, although with potential mutagenic activity. In contrast, chaparrinone (**3**) exhibited more favorable pharmacokinetic characteristics; however, it also showed a higher potential for acute oral toxicity.

Regarding the molecular docking studies, the three quassinoids exhibited favorable interactions with the molecular target’s actin, ALR, and PFOR. Among the compounds analyzed, amarolide (**2**) showed the highest binding affinity toward these proteins, suggesting their possible involvement in its antiparasitic activity. Nevertheless, chaparrinone (**3**) exhibited the highest in vitro antigiardial activity despite showing lower binding affinity. Therefore, its antigiardial activity may be influenced by additional factors, such as cellular permeability, simultaneous interactions with multiple molecular targets, or other mechanisms of parasitic damage.

These results provide initial evidence supporting the potential of quassinoids as bioactive molecules for the development of alternative therapeutic agents against giardiasis, particularly chaparrinone (**3**). Nevertheless, further studies, including enzymatic assays, pharmacokinetic investigations, and in vivo toxicity evaluations, are required to more precisely determine the therapeutic potential of these compounds.

## Figures and Tables

**Figure 1:**
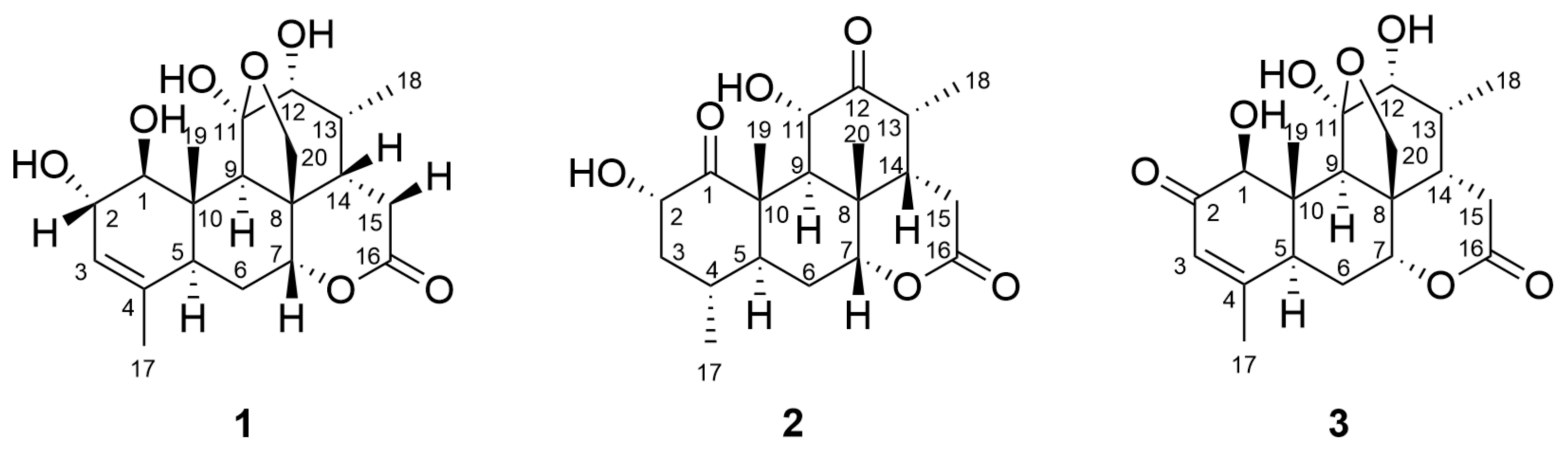
Structures of quassinoids from *Castela tortuosa* Liebm.: chaparrin (**1**), amarolide (**2**), and chaparrinone (**3**).

**Figure 2:**
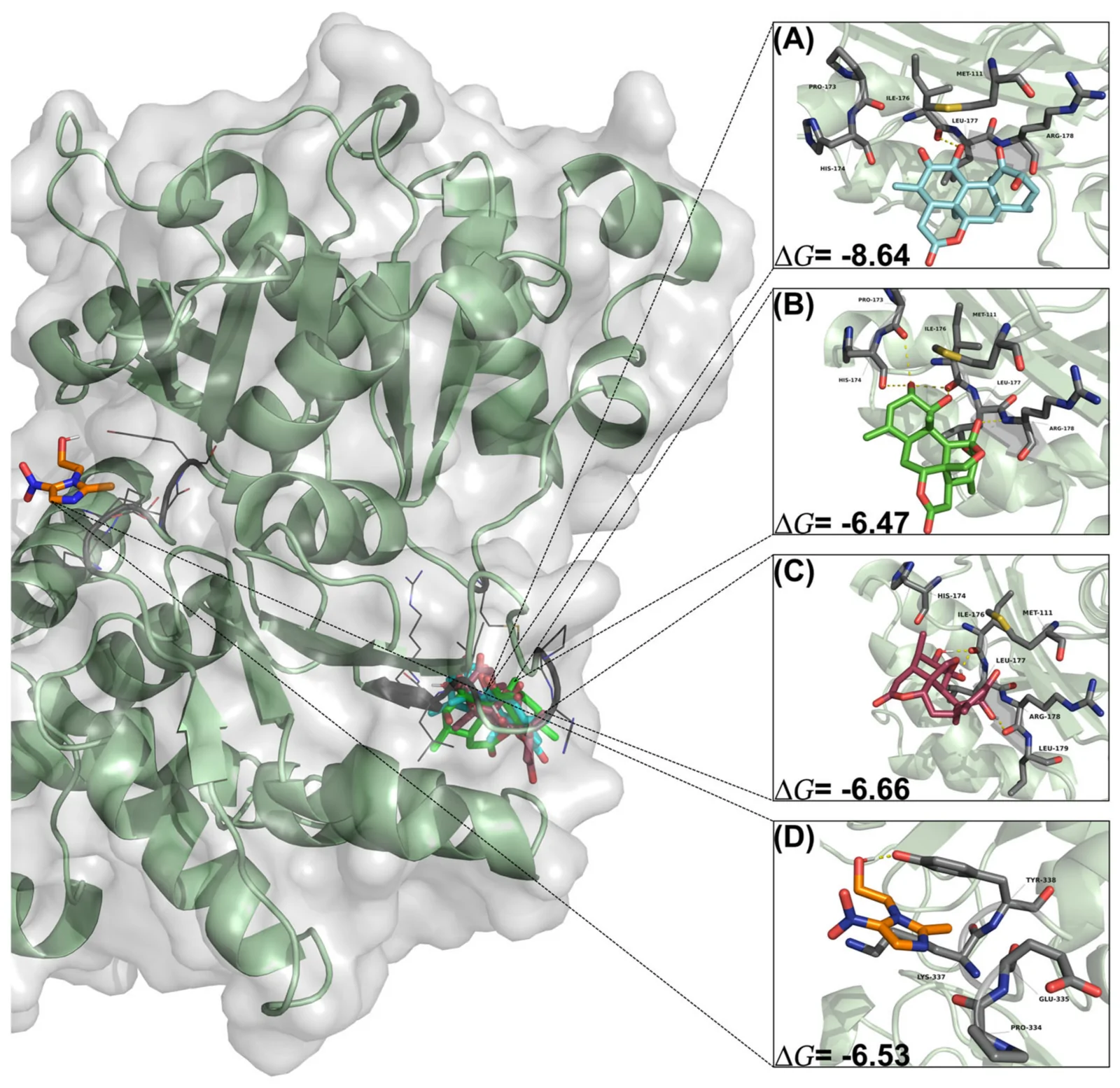
Three-dimensional binding mode and interactions for (**A**) amarolide, (**B**) chaparrin, (**C**) chaparrinone, and (**D**) metronidazole in actin.

**Figure 3:**
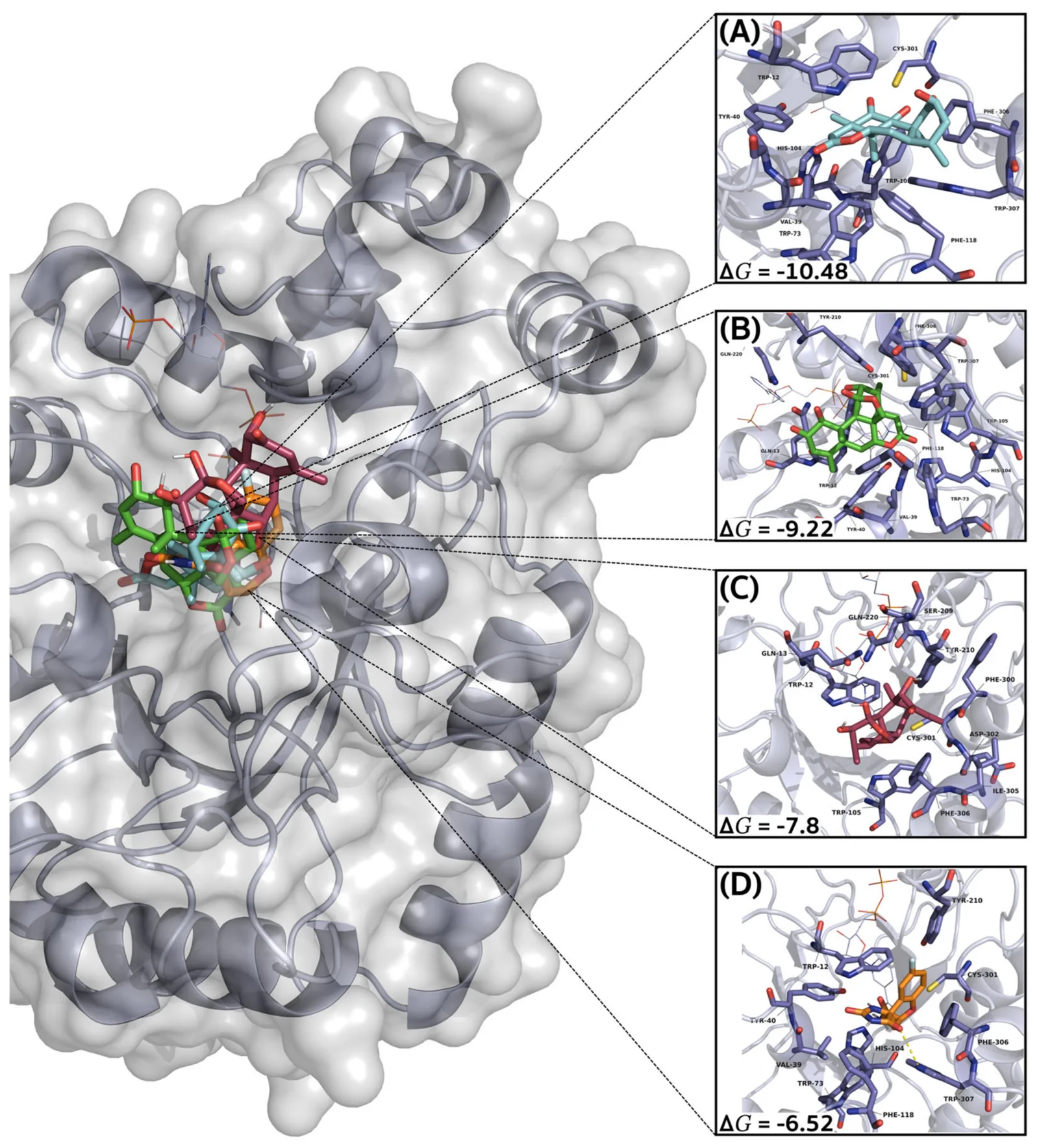
Three-dimensional binding mode and interactions for (**A**) amarolide, (**B**) chaparrin, (**C**) chaparrinone, and (**D**) fidarestat in aldose reductase (ALR).

**Figure 4:**
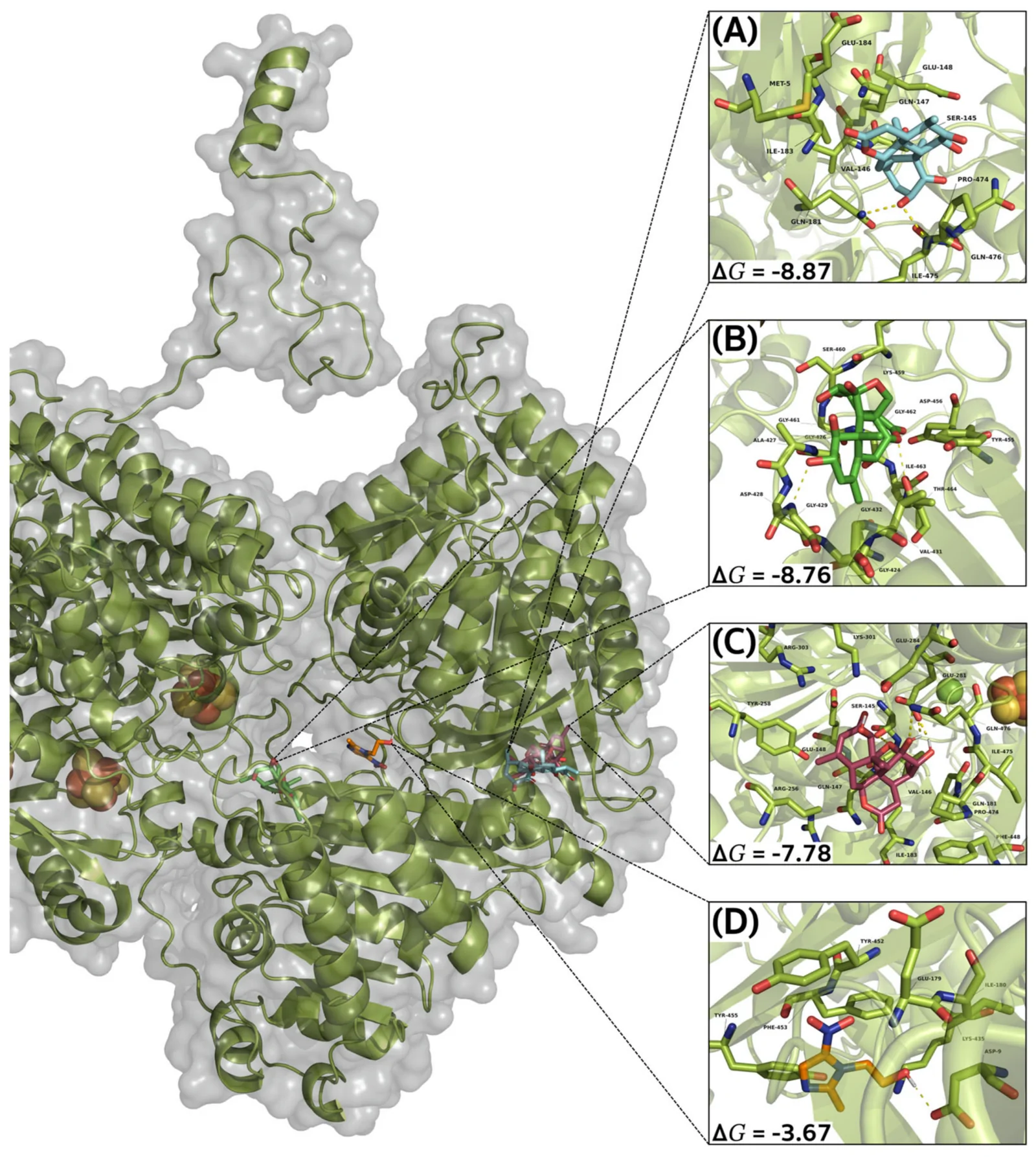
Three-dimensional binding mode and interactions for (**A**) amarolide, (**B**) chaparrin, (**C**) chaparrinone, and (**D**) metronidazole in pyruvate: ferredoxin oxidoreductase (PFOR).

**Table 1:** In vitro antigiardial activity of extracts, active fraction, and pure compounds obtained from *C. tortuosa* Liebm.

Extract/Fraction/Compound	IC_50_ μg mL^−1^ (CI) ^a^ *Giardia lamblia* Wb
Methanolic extract	507.60 (498.50–517.10)
Dichloromethane fraction	67.42 (67.32–67.52)
Chaparrin (**1**)	283.80 (282.60–285.02)
Amarolide (**2**)	126.04 (125.74–126.34)
Chaparrinone (**3**)	18.98 (18.90–19.01)
Metronidazole	0.41 (0.40–0.42)

^a^ Results are expressed as a mean (*n* = 6). CI, 95% confidence interval; correlation coefficient > 0.95.

## Data Availability

The original contributions presented in this study are included in the article/Supplementary Material. Further inquiries can be directed to the corresponding authors.

## Supplementary Materials

The following supporting information can be downloaded at: https://www.mdpi.com/article/10.3390/ijms27198733/s1.
