# Supplementary material for: In Vitro Antigiardial Activity and In Silico Target Exploration of Quassinoids Isolated from Castela tortuosa Liebm
Source: Int J Mol Sci. 2026 Sep 30;27(19):8733. doi: 10.3390/ijms27198733 (PMC13665569; doi:10.3390/ijms27198733)
Supplement: Supplementary file 1 [file ijms-27-08733-s001.zip › ijms-4571941-supplementary.pdf]

Supplementary Information

In Vitro Antigiardial Activity and In Silico Target Exploration of Quassinoids Isolated from *Castela tortuosa* Liebm.

Ulises Murrieta-Dionicio [1], Fernando Calzada [2,*], Elizabeth Barbosa [3], Miguel Valdes [2,3,4], Jesica Ramirez-Santos [2,3], Benito Reyes-Trejo [1,*], Holber Zuleta-Prada [1] and Lino Reyes [5]

[1] Laboratorio de Productos Naturales, Área de Química, Departamento de Preparatoria Agrícola, Universidad Autónoma Chapingo, Chapingo 56230, Mexico; murrieta.dionicio.091293@gmail.com (U.M.-D.); hzuletap@chapingo.mx (H.Z.-P.)

[2] Unidad de Investigación Médica en Farmacología, UMAE Hospital de Especialidades, 2° Piso CORSE Centro Médico Nacional Siglo XXI, IMSS, Ciudad de Mexico 06720, Mexico; valdesguevaramiguel@gmail.com (M.V.); jes.ram.san@gmail.com (J.R.-S.)

[3] Escuela Superior de Medicina, Instituto Politécnico Nacional, Ciudad de Mexico 07738, Mexico; rebc78@yahoo.com.mx

[4] Laboratorio de Inmunología, Departamento de Sistemas Biológicos, Universidad Autónoma Metropolitana Unidad Xochimilco, Calz. del Hueso 1100, Coapa, Villa Quietud, Coyoacán, Ciudad de Mexico 04960, Mexico

[5] Departamento de Química Orgánica, Facultad de Química, Universidad Nacional Autónoma de México, Ciudad Universitaria, Delegación Coyoacán 04510, Mexico; linoj23@quimica.unam.mx

* Correspondence: fercalber10@gmail.com (F.C.); breyest@chapingo.mx (B.R.-T.)

Contents

Supplementary Figures

Figure S1. ATR-FTIR spectrum of compound **1**. 4
Figure S2. ^{13}C NMR spectrum of compound **1** in pyridine-d_5 at 298 K (100 MHz). 4
Figure S3. ^{1}H NMR spectrum of compound **1** in pyridine-d_5 at 298 K (400 MHz). 5
Figure S4. ^{1}H–^{13}C HSQC spectrum of compound **1** in pyridine-d_5 at 298 K (400 MHz). 6
Figure S5. ^{1}H–^{1}H COSY spectrum of compound **1** in pyridine-d_5 at 298 K (400 MHz). 7
Figure S6. ^{1}H–^{13}C HMBC spectrum of compound **1** in pyridine-d_5 at 298 K (400 MHz). 8
Figure S7. ^{13}C NMR spectrum of compound **1** in DMSO-d_6 at 298 K (100 MHz). 8
Figure S8. ^{1}H NMR spectrum of compound **1** in DMSO-d_6 at 298 K (400 MHz). 9
Figure S9. ^{1}H–^{13}C HSQC spectrum of compound **1** in DMSO-d_6 at 298 K (400 MHz). 10
Figure S10. ^{1}H–^{1}H COSY spectrum of compound **1** in DMSO-d_6 at 298 K (400 MHz). 11
Figure S11. ^{1}H–^{13}C HMBC spectrum of compound **1** in DMSO-d_6 at 298 K (400 MHz). 12
Figure S12. ATR-FTIR spectrum of compound **2**. 14
Figure S13. ^{13}C NMR spectrum of compound **2** in pyridine-d_5 at 298 K (100 MHz). 14
Figure S14. ^{1}H NMR spectrum of compound **2** in pyridine-d_5 at 298 K (400 MHz). 15
Figure S15. ^{1}H–^{13}C HSQC spectrum of compound **2** in pyridine-d_5 at 298 K (400 MHz). 16
Figure S16. ^{1}H–^{1}H COSY spectrum of compound **2** in pyridine-d_5 at 298 K (400 MHz). 17
Figure S17. ^{1}H–^{13}C HMBC spectrum of compound **2** in pyridine-d_5 at 298 K (400 MHz). 18
Figure S18. ^{13}C NMR spectrum of compound **2** in $CDCl_3$ at 298 K (100 MHz). 18
Figure S19. ^{1}H NMR spectrum of compound **2** in $CDCl_3$ at 298 K (400 MHz). 19
Figure S20. ^{1}H–^{13}C HSQC spectrum of compound **2** in $CDCl_3$ at 298 K (400 MHz). 20
Figure S21. ^{1}H–^{1}H COSY spectrum of compound **2** in $CDCl_3$ at 298 K (400 MHz). 21
Figure S22. ^{1}H–^{13}C HMBC spectrum of compound **2** in $CDCl_3$ at 298 K (400 MHz). 22
Figure S23. ATR-FTIR spectrum of compound **3**. 24
Figure S24. ^{13}C NMR spectrum of compound **3** in DMSO-d_6 at 298 K (100 MHz). 24
Figure S25. ^{1}H NMR spectrum of compound **3** in DMSO-d_6 at 298 K (400 MHz). 25
Figure S26. ^{1}H–^{13}C HSQC spectrum of compound **3** in DMSO-d_6 at 298 K (400 MHz). 26
Figure S27. ^{1}H–^{1}H COSY spectrum of compound **3** in DMSO-d_6 at 298 K (400 MHz). 27
Figure S28. ^{1}H–^{13}C HMBC spectrum of compound **3** in DMSO-d_6 at 298 K (400 MHz). 28

Supplementary Tables

Table S1. ^{1}H and ^{13}C data of compound **1**. 13

Table S2. ^{1}H and ^{13}C data of compound **2**. 23
Table S3. ^{1}H and ^{13}C data of compound **3**. 29
Table S4. Physicohemical, pharmacokinetic, and toxicologic predictive values of quassinoids and metronidazole[a]... 30

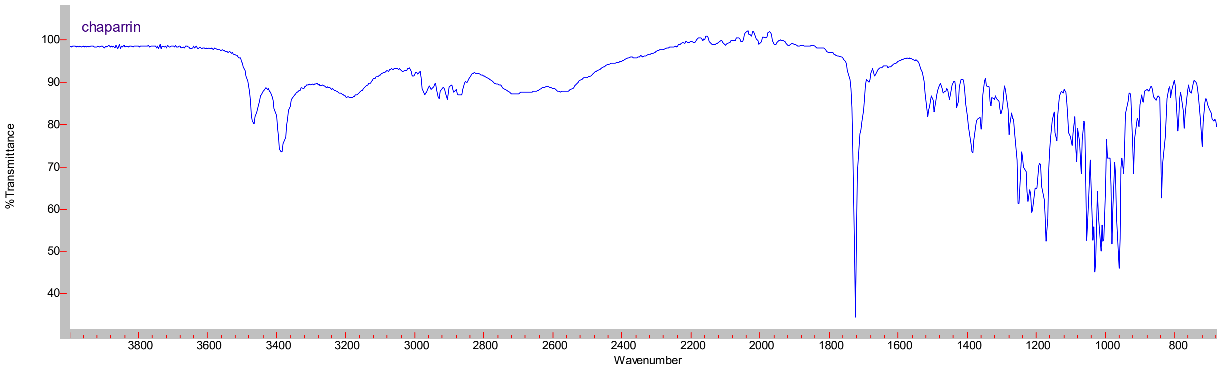

Figure S1. ATR-FTIR spectrum of compound **1**.

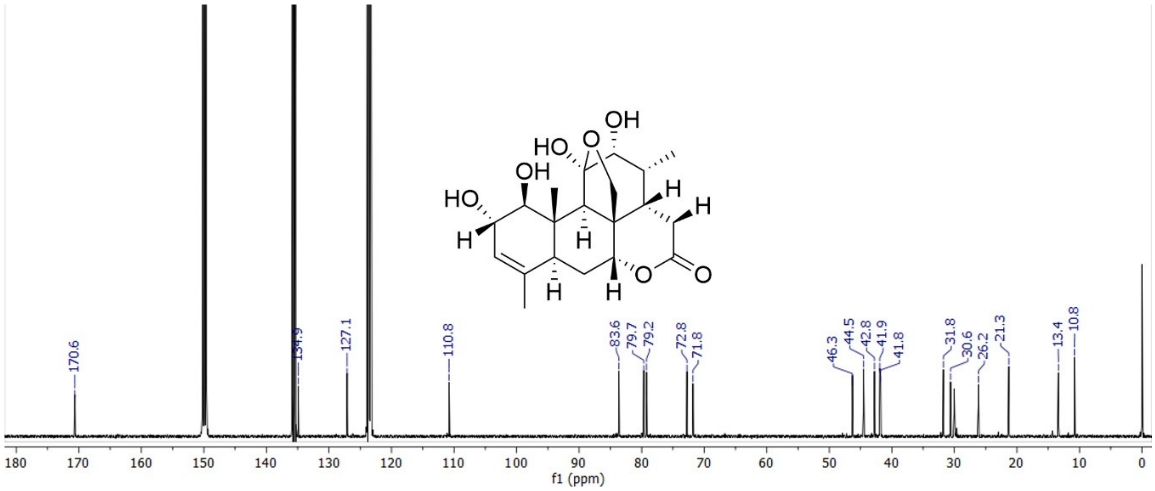

Figure S2. ^{13}C NMR spectrum of compound **1** in pyridine-d_5 at 298 K (100 MHz).

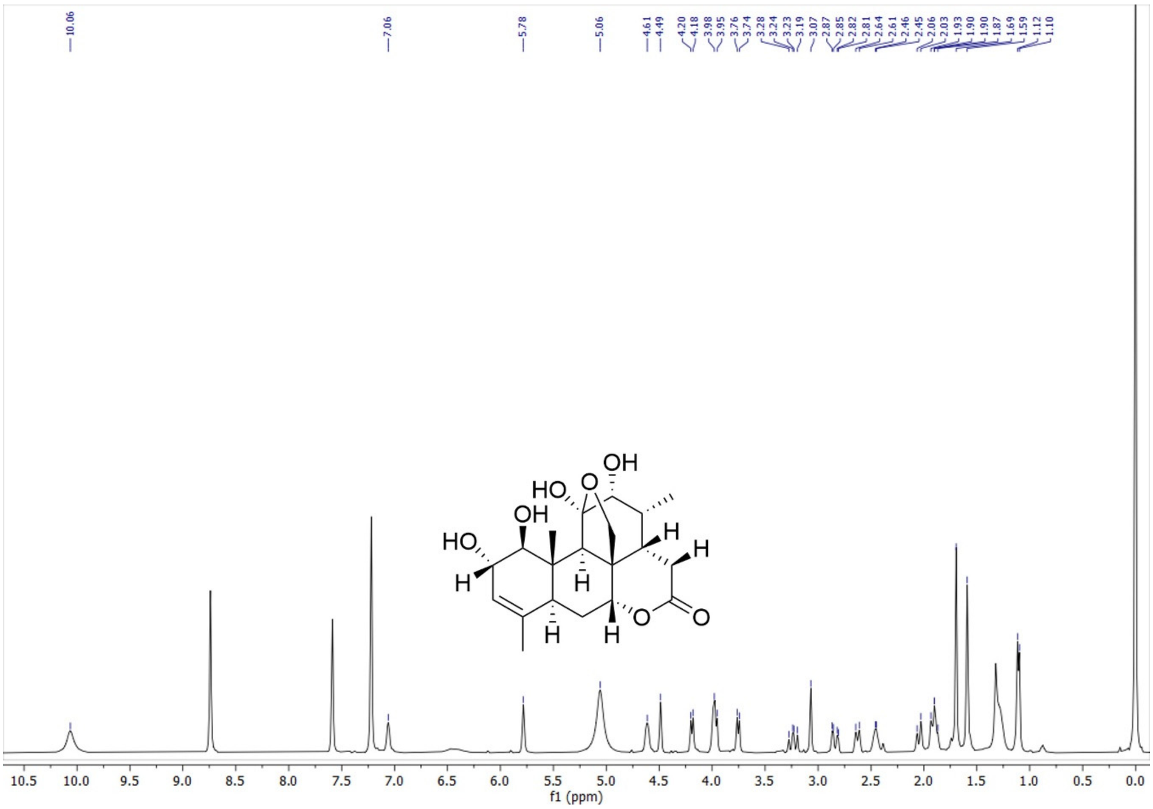

Figure S3. ^{1}H NMR spectrum of compound **1** in pyridine-d_5 at 298 K (400 MHz).

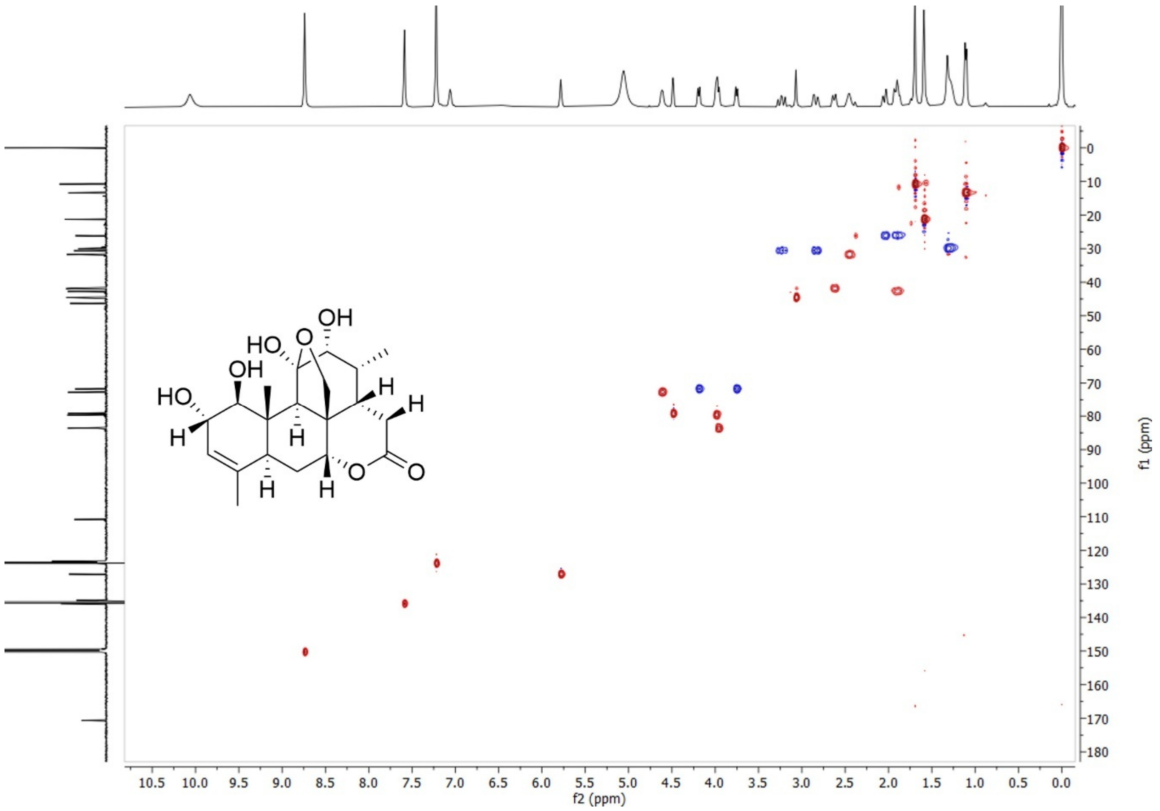

Figure S4. ^{1}H–^{13}C HSQC spectrum of compound **1** in pyridine-d_5 at 298 K (400 MHz).

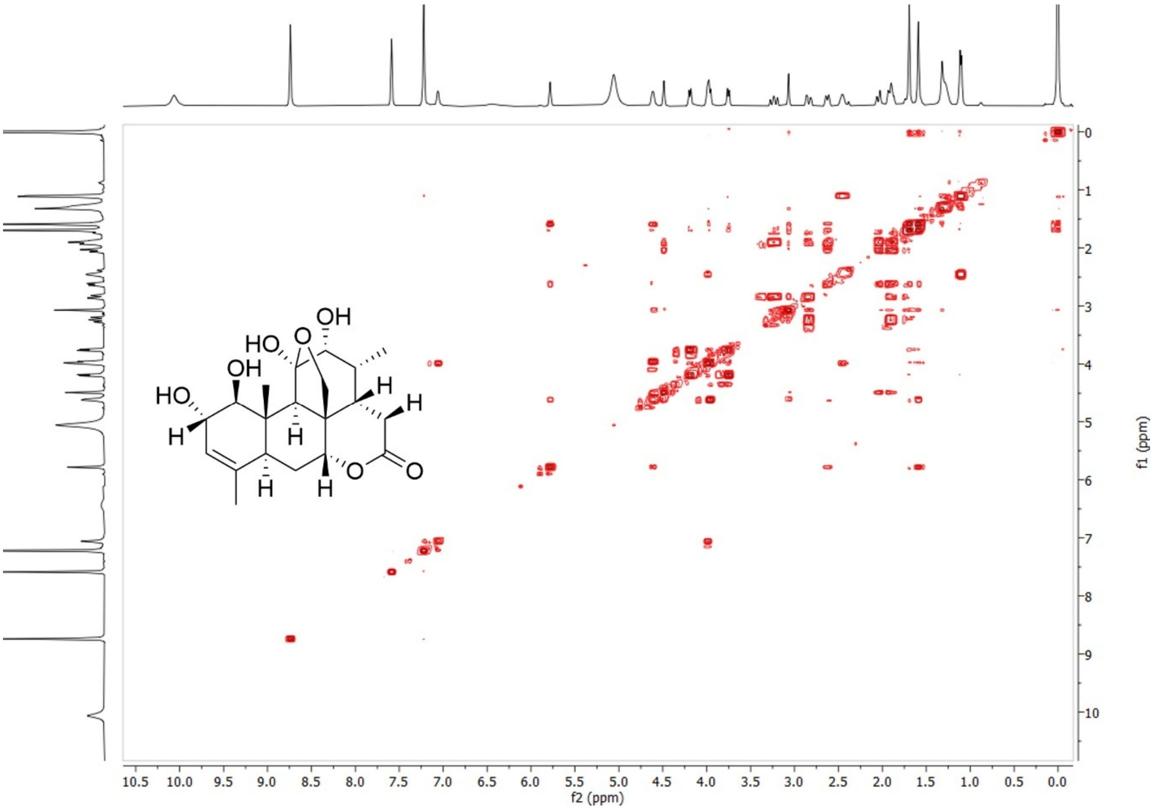

Figure S5. ^1H–^1H COSY spectrum of compound **1** in pyridine-d_5 at 298 K (400 MHz).

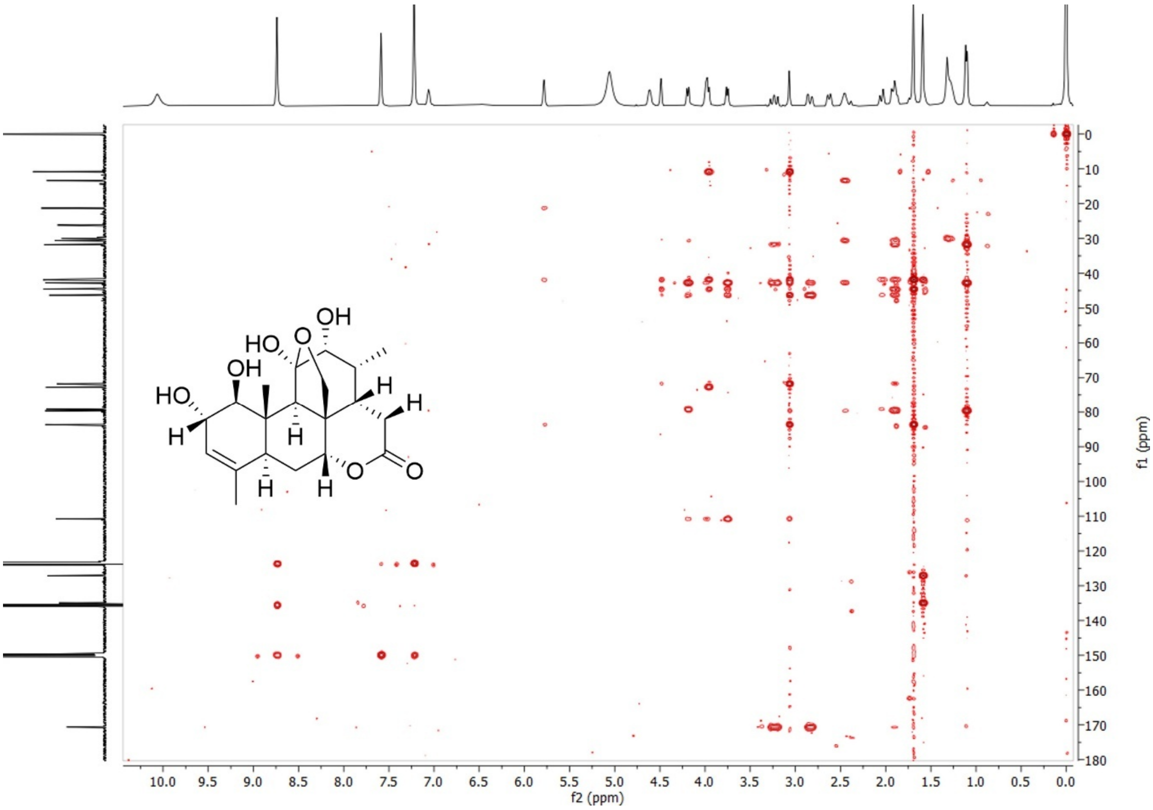

Figure S6. ^{1}H–^{13}C HMBC spectrum of compound **1** in pyridine-d_5 at 298 K (400 MHz).

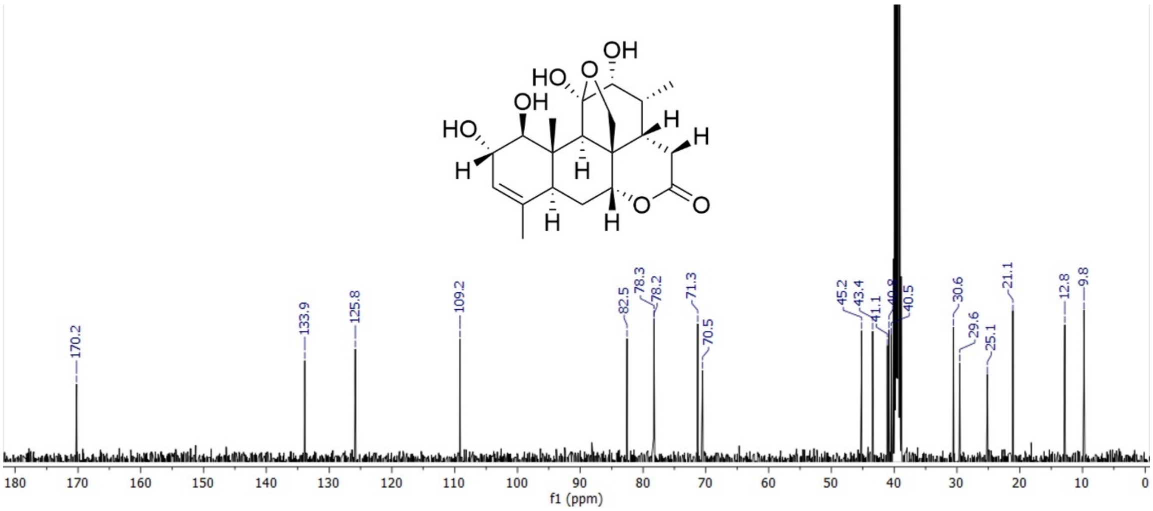

Figure S7. ^{13}C NMR spectrum of compound **1** in DMSO-d_6 at 298 K (100 MHz).

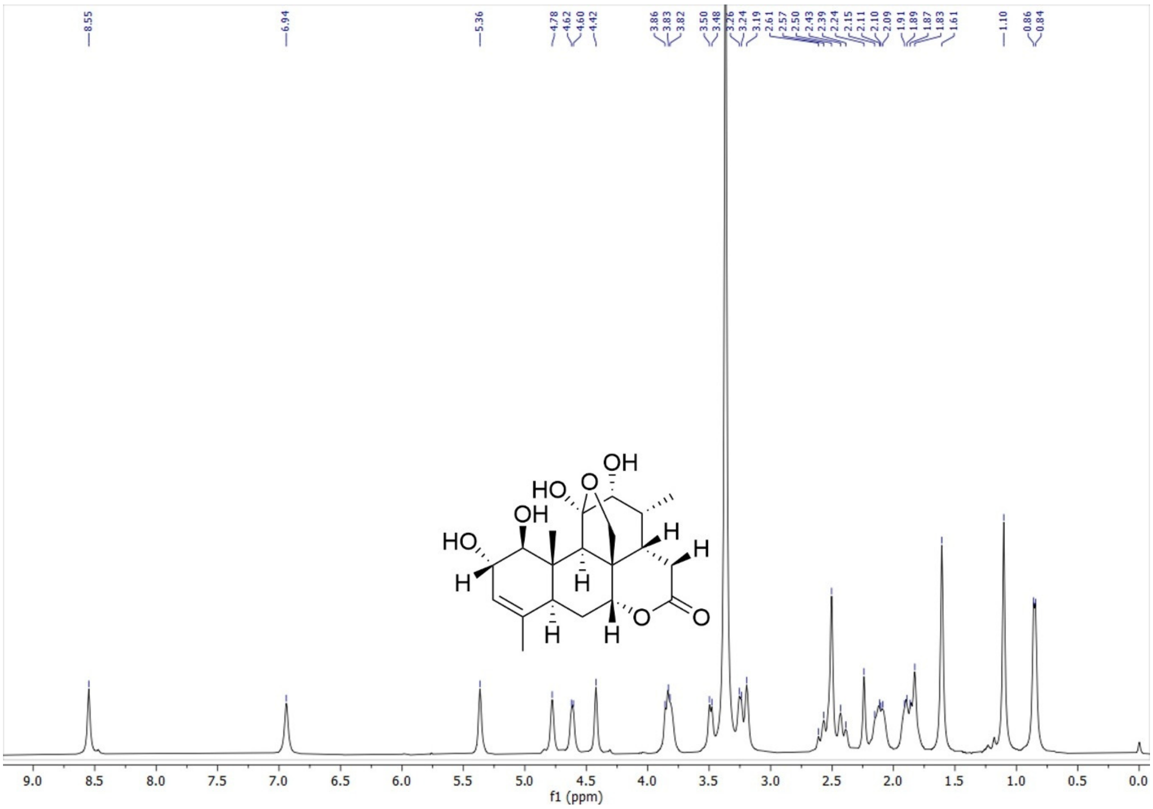

Figure S8. ^{1}H NMR spectrum of compound **1** in DMSO-d_6 at 298 K (400 MHz).

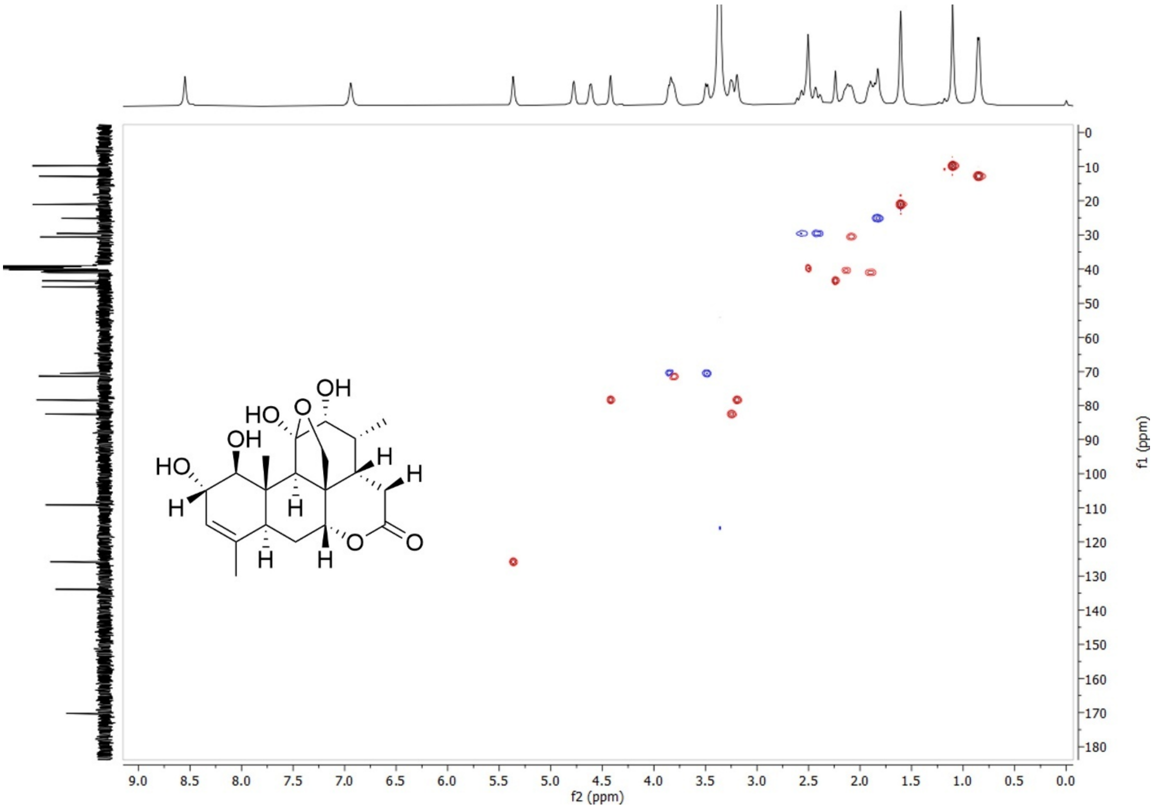

Figure S9. ^{1}H–^{13}C HSQC spectrum of compound **1** in DMSO-d_6 at 298 K (400 MHz).

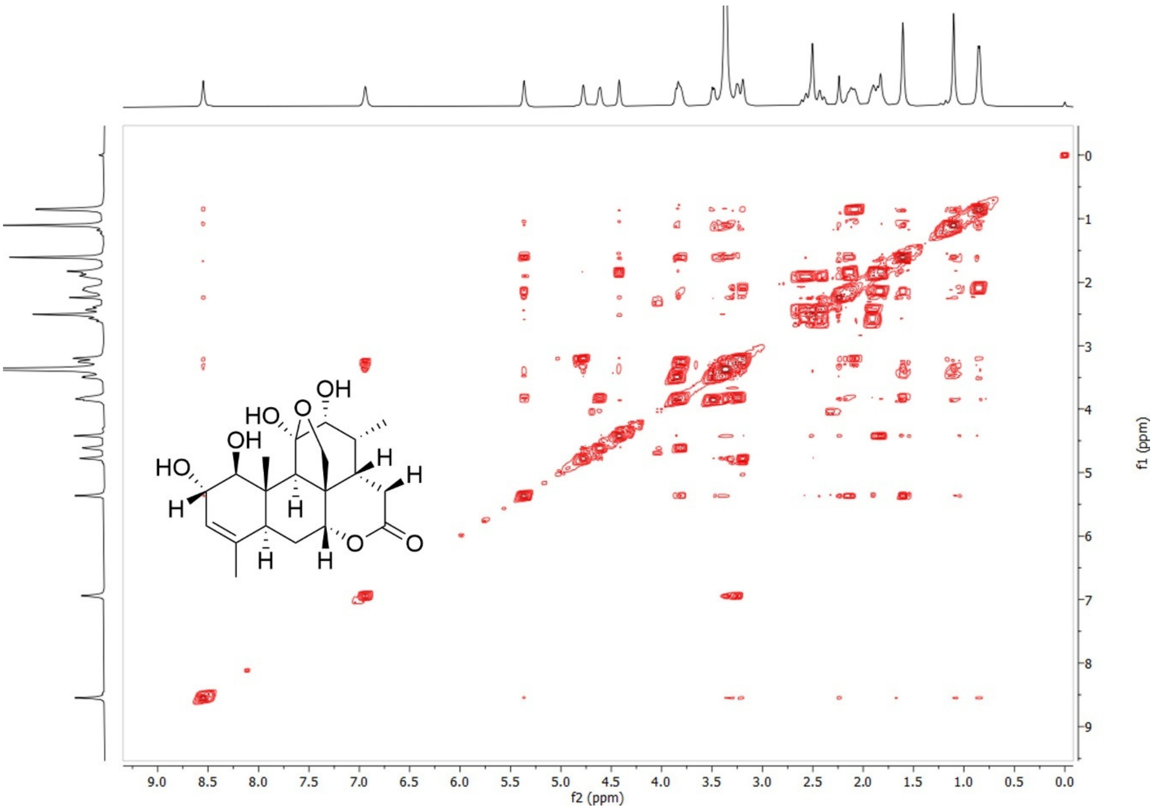

Figure S10. ^1H–^1H COSY spectrum of compound **1** in DMSO-d_6 at 298 K (400 MHz).

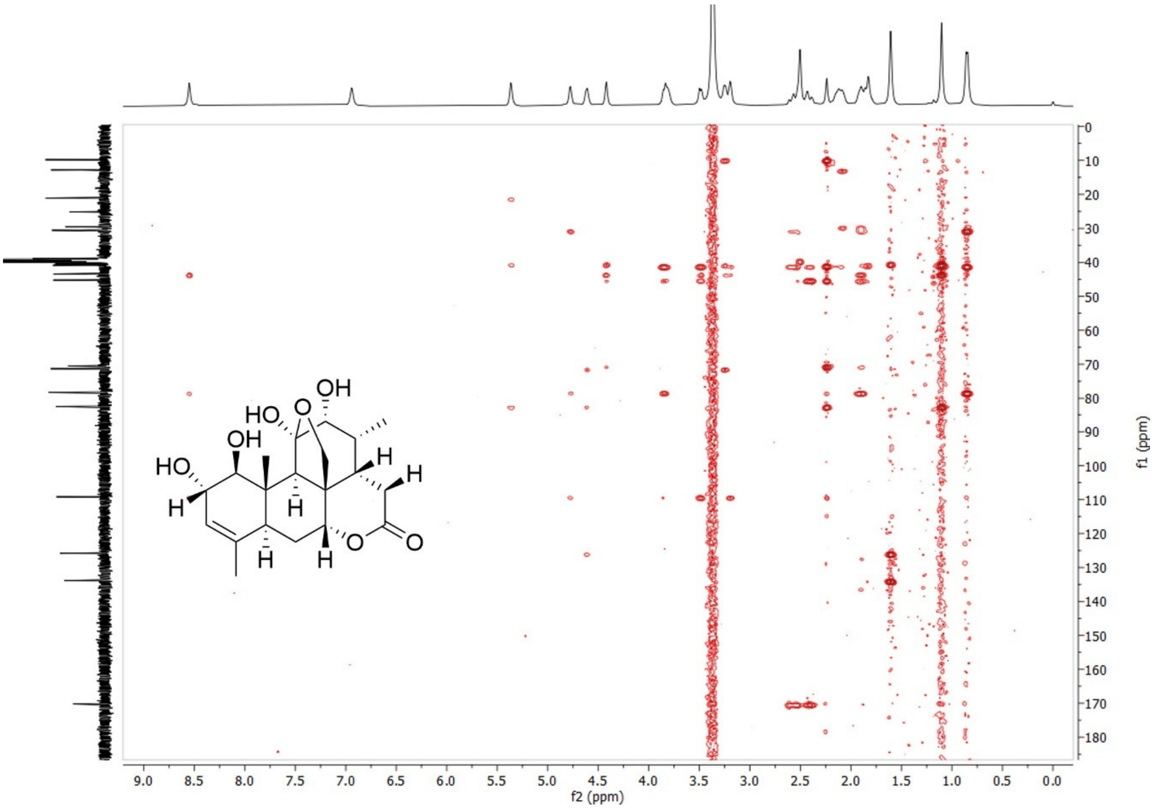

Figure S11. ^{1}H–^{13}C HMBC spectrum of compound **1** in DMSO-d_6 at 298 K (400 MHz).

Table S1. ^{1}H and ^{13}C data of compound **1**.

Position	Compound **1** (pyridine-d_5)		Compound **1** (DMSO-d_6)	
	δ_C	δ_H, mult. (J in Hz)	δ_C	δ_H, mult. (J in Hz)
1	83.6	3.95, s	82.5	3.25, d (7.3)
2	72.8	4.61, s	71.3	3.82, s
3	127.1	5.78, s	125.8	5.36, s
4	134.9	-	133.9	-
5	41.9	2.63, d (13.1)	40.5	2.13, d (16.1)
6	26.2	2.05, d (14.5)	25.1	1.85, d (15.8)
		1.90, m		
7	79.2	4.49, s	78.2	4.42, s
8	46.3	-	45.2	-
9	44.5	3.07, s	43.4	2.24, s
10	41.8	-	40.8	-
11	110.8	-	109.2	-
12	79.7	3.98, s	78.3	3.19, s
13	31.8	2.46, s	30.6	2.09, m
14	42.8	1.90, m	41.1	1.89, s
15	30.6	3.23, m	29.6	2.41, d (17.6)
		2.84, m		2.59, d (17.9)
16	170.6	-	170.2	-
17	21.3	1.59, s	21.1	1.61, s
18	13.4	1.11, d (7.1)	12.8	0.85, d (7.0)
19	10.8	1.69, s	9.8	1.10, s
20	71.8	3.75, d (8.3)	70.5	3.49, d (8.5)
		4.19, d (8.4)		3.86, d (8.5)
1-OH		7.06, s		6.94, s
2-OH		5.06, s		4.61, s
11-OH		10.06, s		8.55, s
12-OH		5.06, s		4.78, s

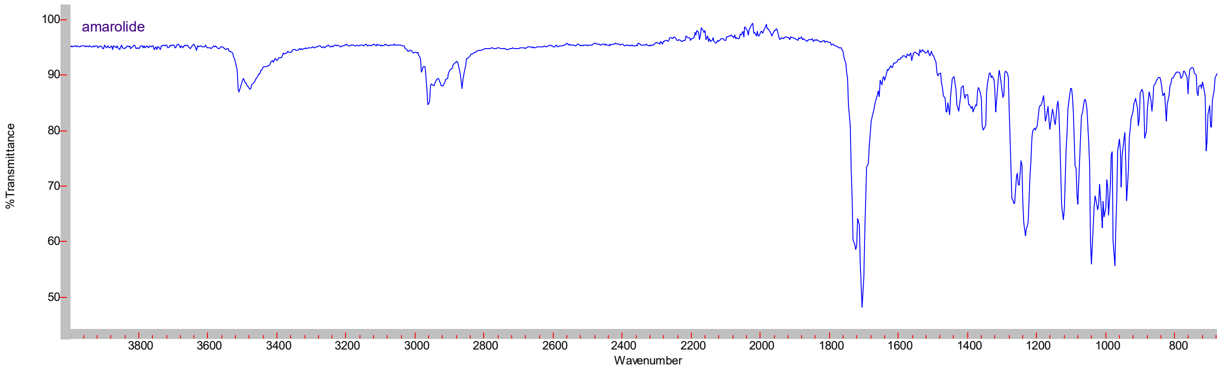

Figure S12. ATR-FTIR spectrum of compound **2**.

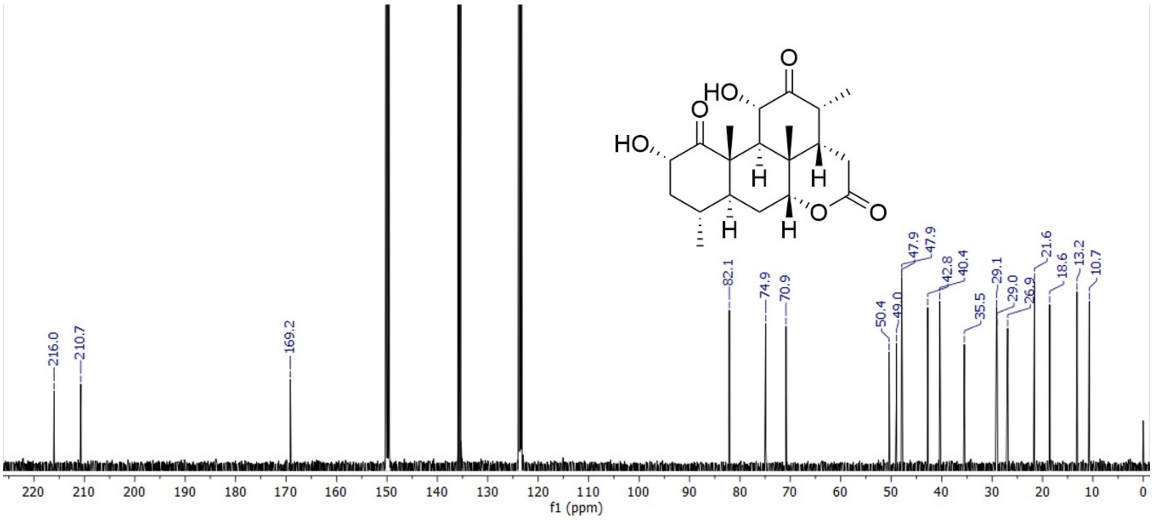

Figure S13. ^{13}C NMR spectrum of compound **2** in pyridine-d_5 at 298 K (100 MHz).

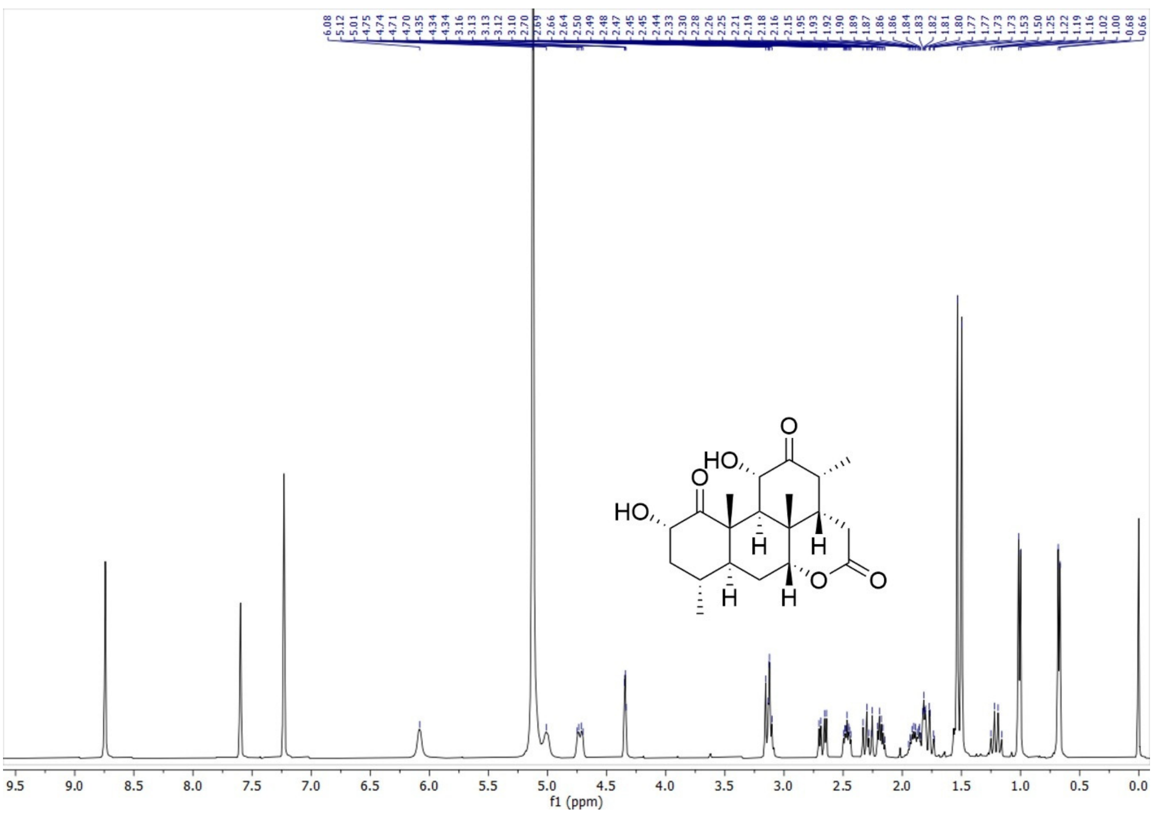

Figure S14. ^{1}H NMR spectrum of compound **2** in pyridine-d_5 at 298 K (400 MHz).

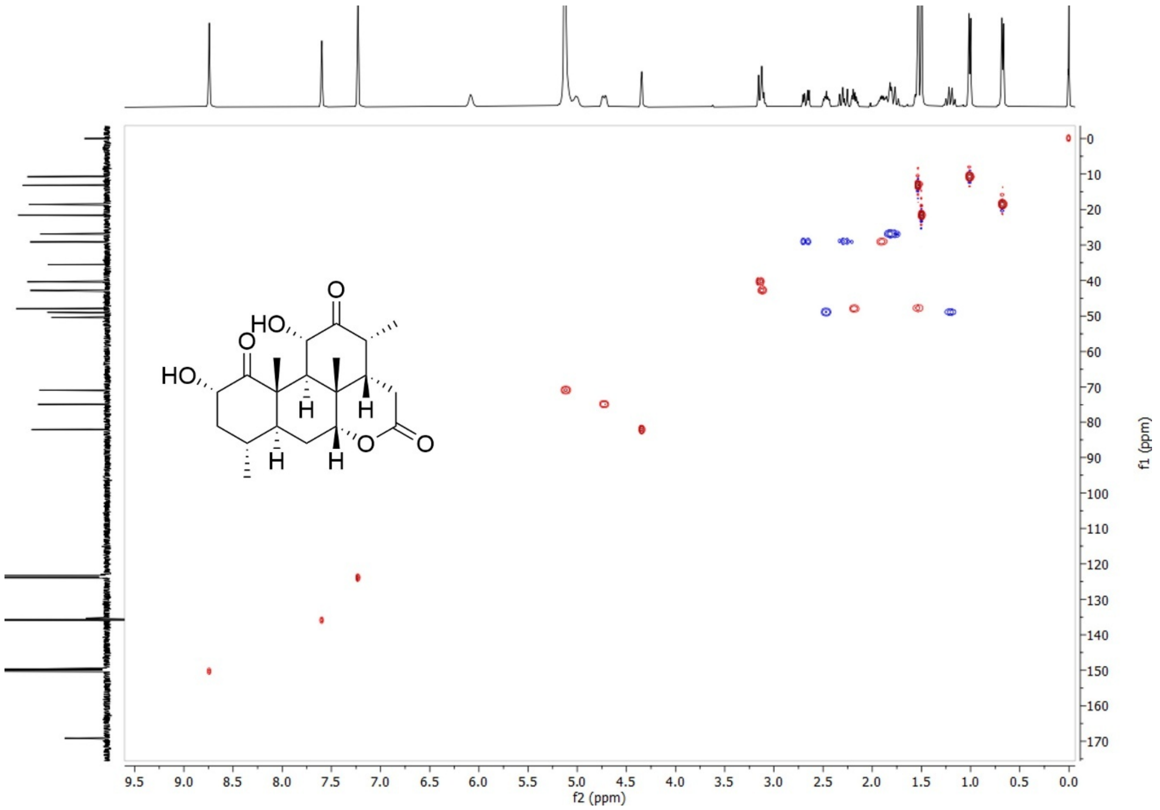

Figure S15. ^1H–^{13}C HSQC spectrum of compound **2** in pyridine-d_5 at 298 K (400 MHz).

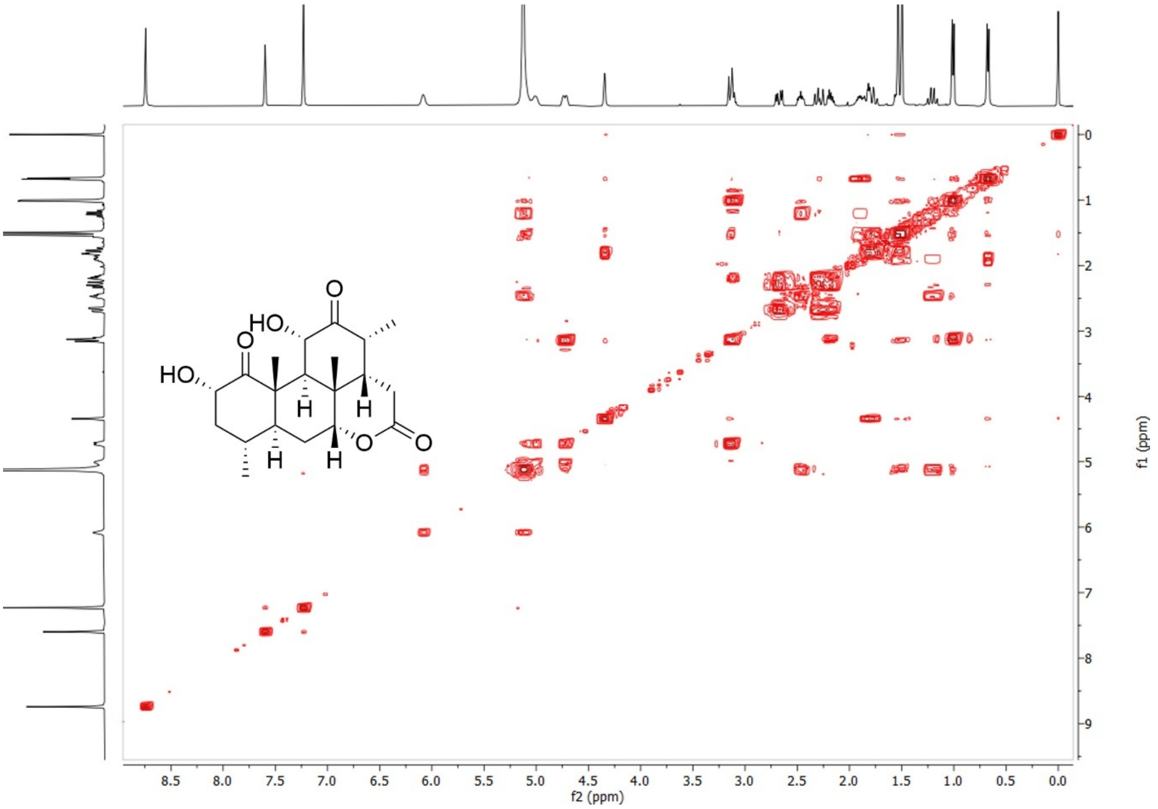

Figure S16. ^{1}H–^{1}H COSY spectrum of compound **2** in pyridine-d_5 at 298 K (400 MHz).

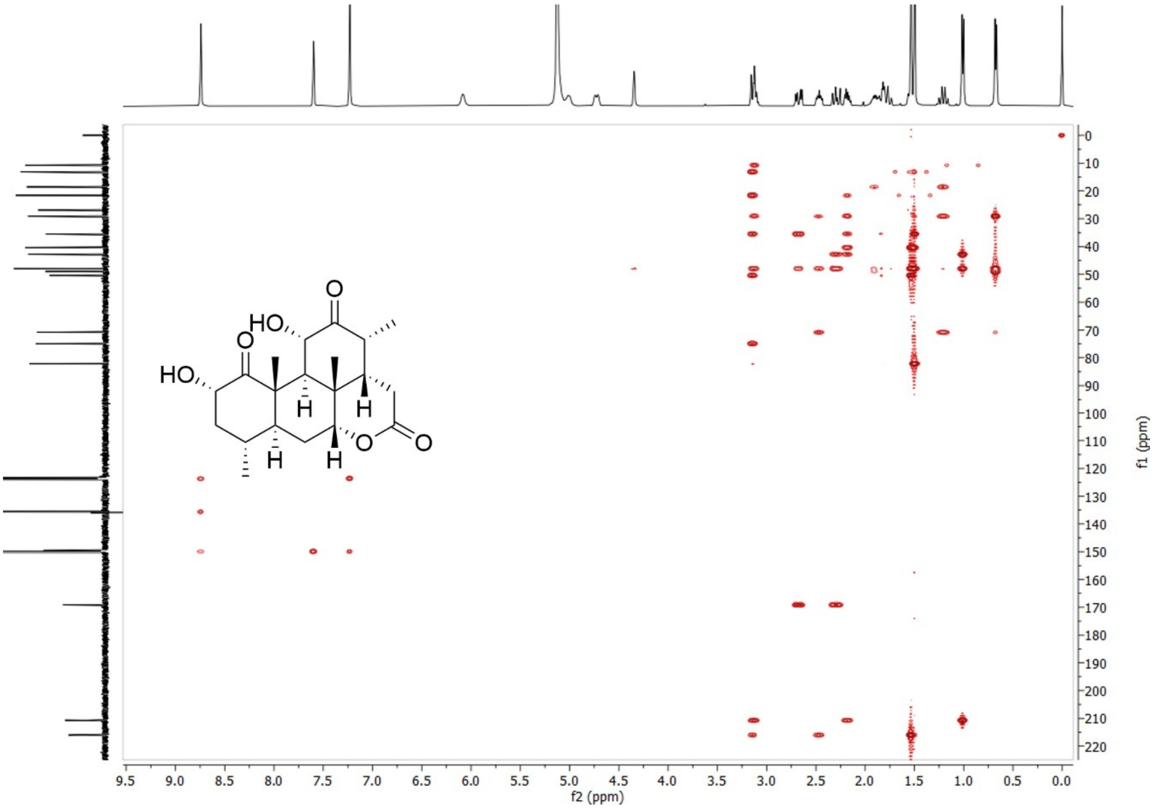

Figure S17. ^{1}H–^{13}C HMBC spectrum of compound **2** in pyridine-d_5 at 298 K (400 MHz).

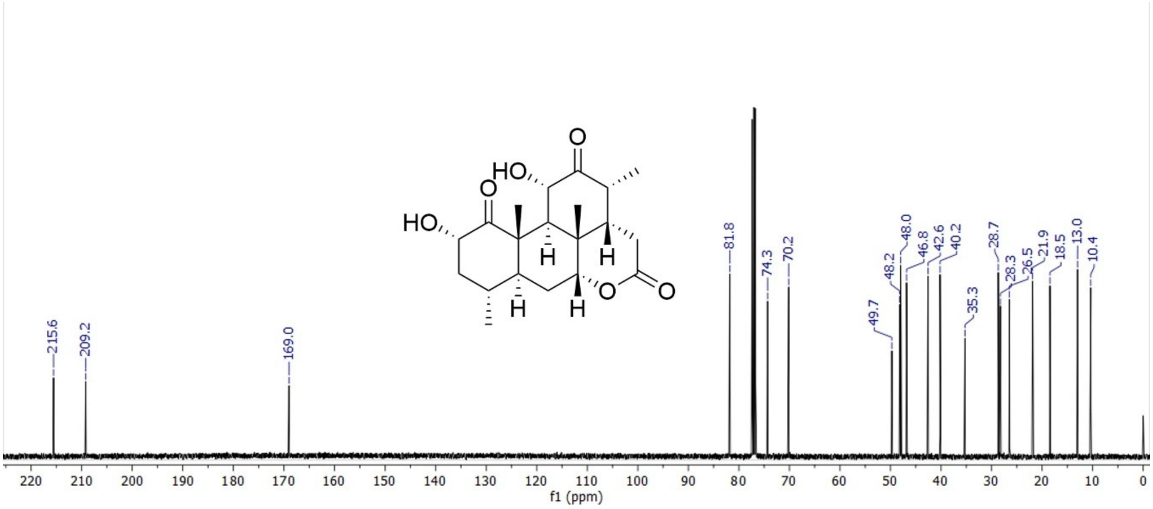

Figure S18. ^{13}C NMR spectrum of compound **2** in $CDCl_3$ at 298 K (100 MHz).

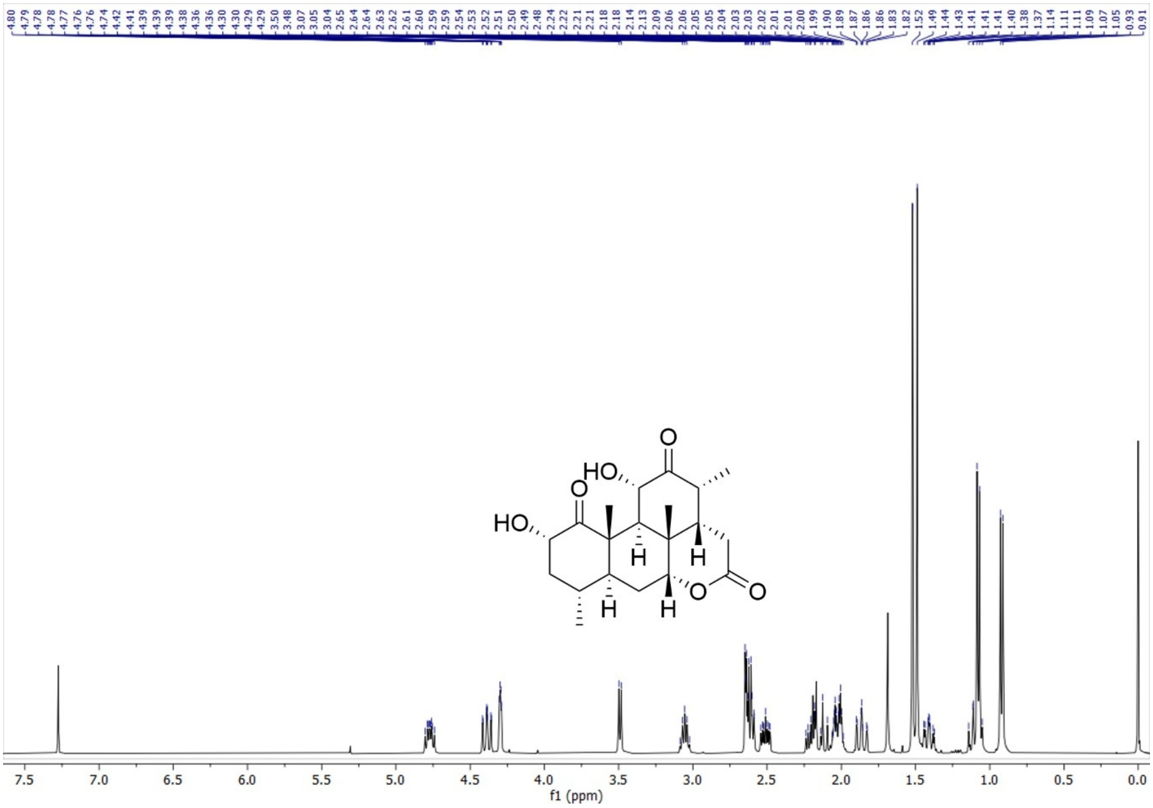

Figure S19. ^{1}H NMR spectrum of compound **2** in $CDCl_3$ at 298 K (400 MHz).

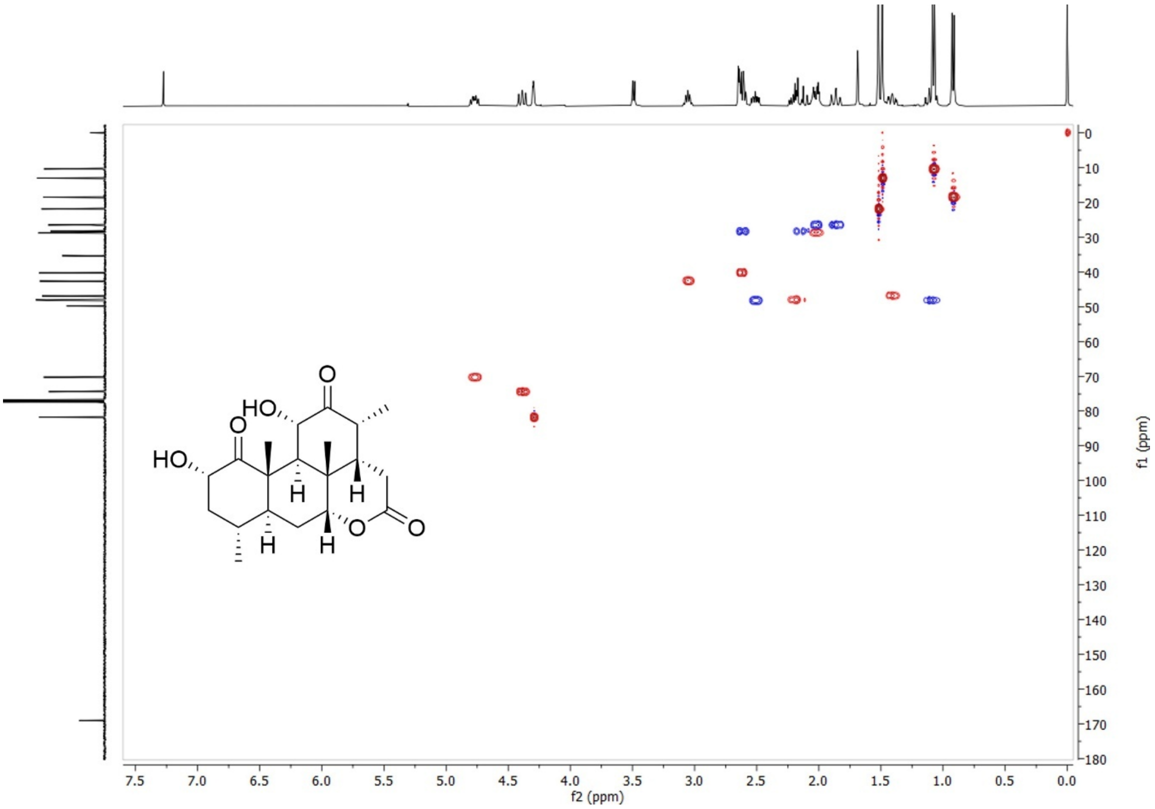

Figure S20. ^{1}H–^{13}C HSQC spectrum of compound **2** in $CDCl_3$ at 298 K (400 MHz).

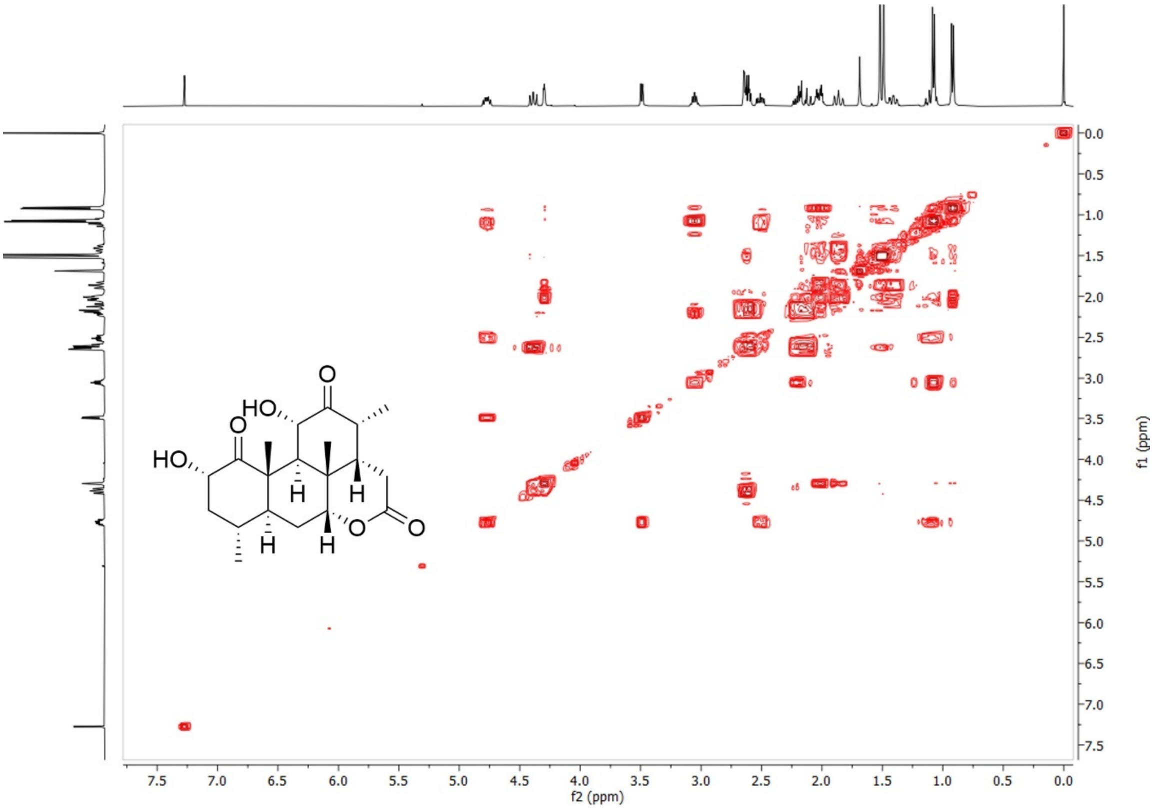

Figure S21. ^{1}H–^{1}H COSY spectrum of compound **2** in $CDCl_3$ at 298 K (400 MHz).

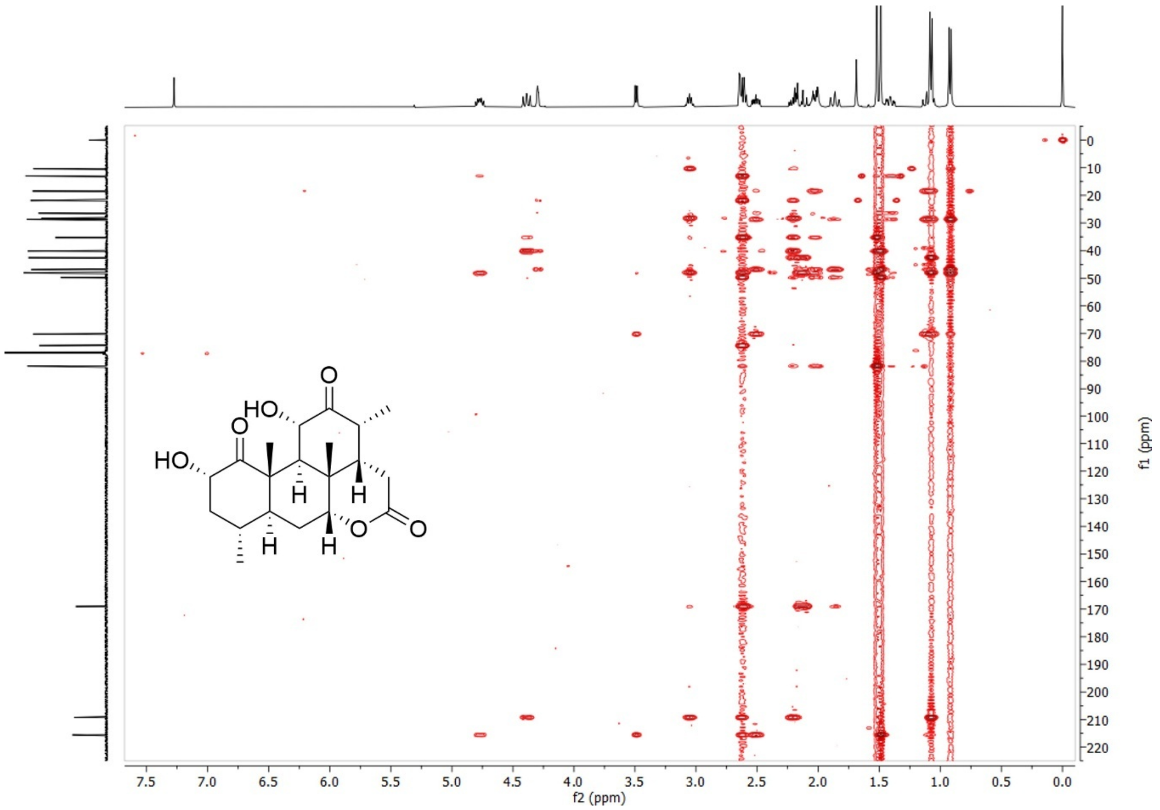

Figure S22. ^{1}H–^{13}C HMBC spectrum of compound **2** in $CDCl_3$ at 298 K (400 MHz).

Table S2. ^{1}H and ^{13}C data of compound **2**.

Position	Compound **2** (pyridine-d_5)		Compound **2** ($CDCl_3$)	
	δ_C	δ_H, mult. (J in Hz)	δ_C	δ_H, mult. (J in Hz)
1	216.0	-	215.6	-
2	70.9	5.12,	70.2	4.77, ddd (11.1, 7.8, 6.1)
3	49.0	2.47, ddd (12.3, 7.6, 4.3)	48.2	2.51, ddd (12.6, 7.8, 4.3)
		1.20, q (12.1)		1.09,
4	29.12	1.89, m	28.7	2.03, m
5	47.91	1.53, m	46.8	1.41, ddd (12.8, 11.2, 3.6)
6	26.9	1.76, m	26.5	1.86, ddd (14.8, 12.9, 2.1)
		1.82, m		2.03, m
7	82.1	4.34, m	81.8	4.30, dd (3.7, 2.1)
8	35.5	-	35.3	-
9	40.4	3.14, d (12.9)	40.2	2.64, dd (4.4, 2.3)
10	50.4	-	49.7	-
11	74.9	4.73, m	74.3	4.39, ddd (12.4, 10.2, 1.1)
12	210.7	-	209.2	-
13	42.8	3.11, m	42.6	3.05, p (6.6)
14	47.9	2.18, dt (12.2, 6.0)	48.0	2.19, m
15	29.0	2.29, m	28.3	2.14, m
		2.67, dd (12.5, 6.8)		2.61, dd (8.1, 5.3)
16	169.2	-	169.0	-
17	18.6	0.67, d (6.4)	18.5	0.92, d (6.5)
18	10.7	1.01, d (6.8)	10.4	1.08, d (6.6)
19	13.2	1.53, s	13.0	1.49, s
20	21.6	1.50, s	21.9	1.52, s
2-OH		6.08, s		3.49, (6.4)
11-OH				

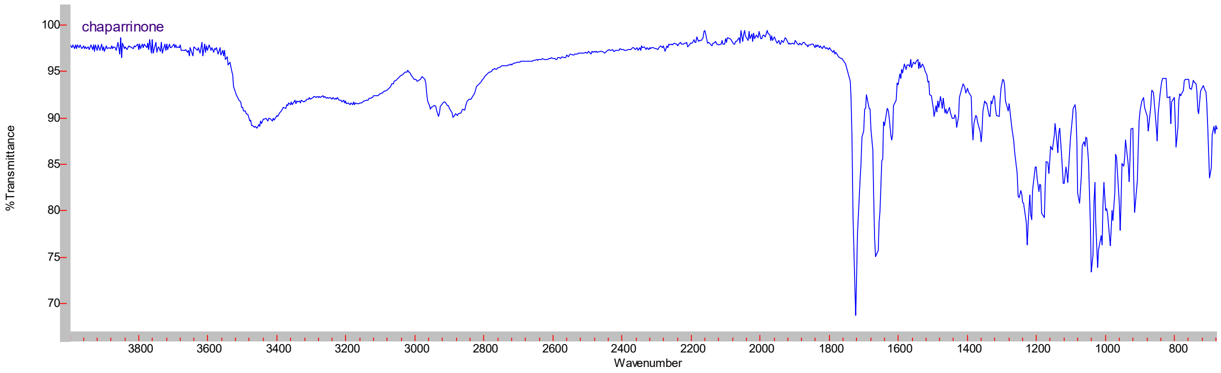

Figure S23. ATR-FTIR spectrum of compound **3**.

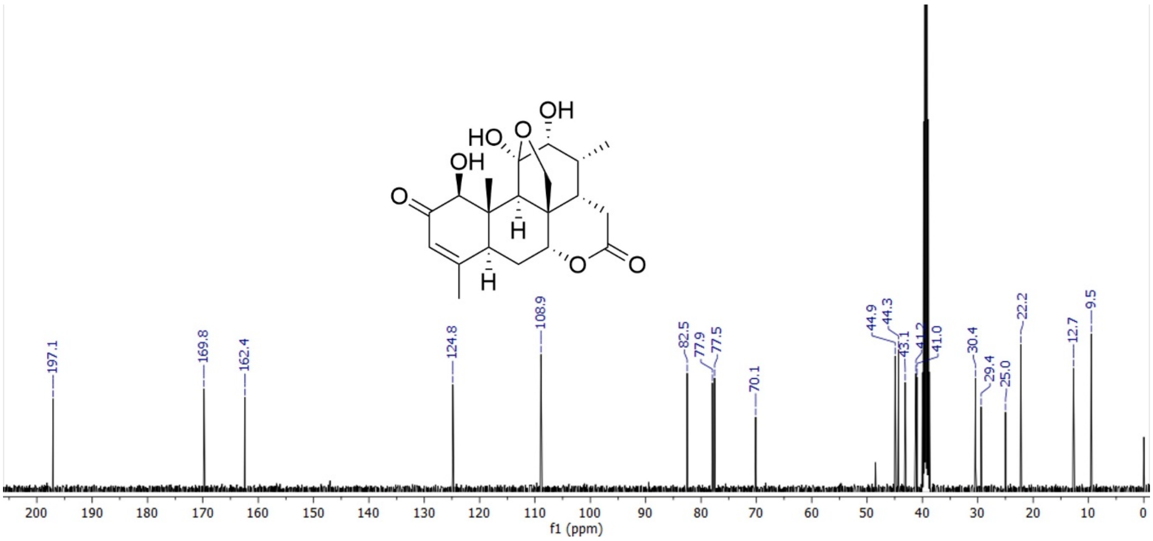

Figure S24. ^{13}C NMR spectrum of compound **3** in DMSO-d_6 at 298 K (100 MHz).

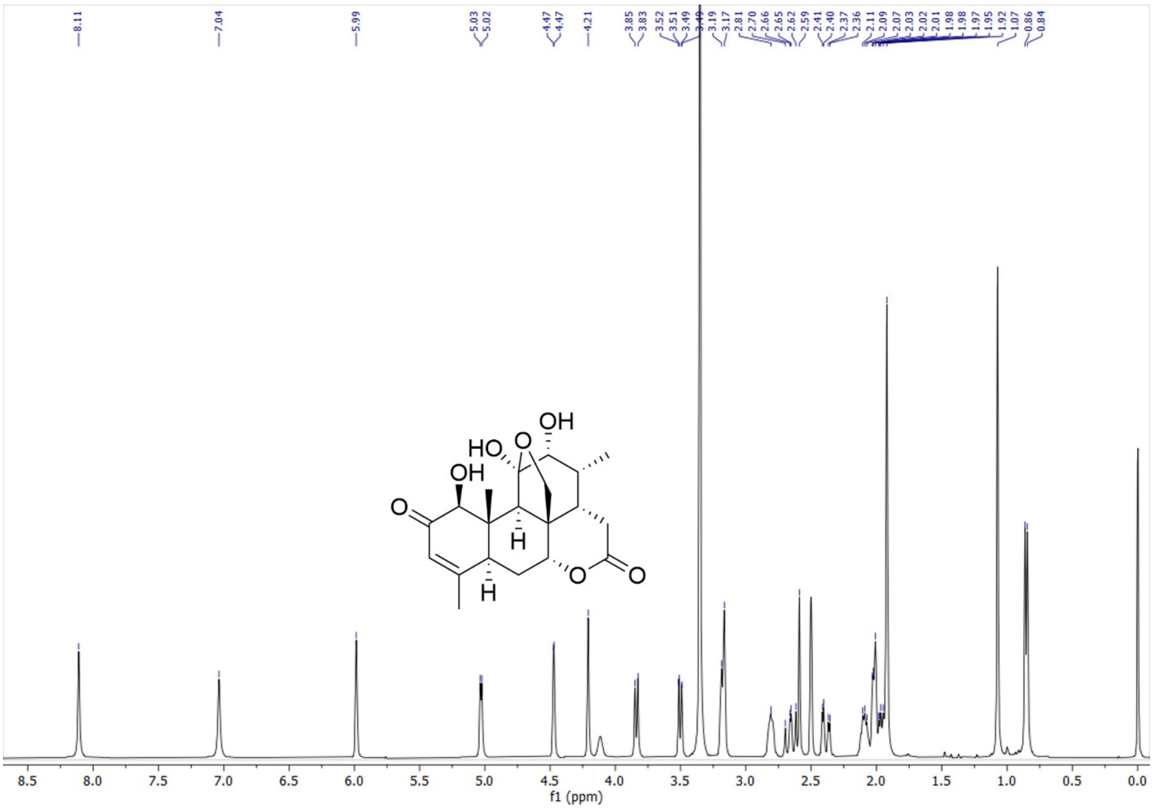

Figure S25. ^{1}H NMR spectrum of compound **3** in DMSO-d_6 at 298 K (400 MHz).

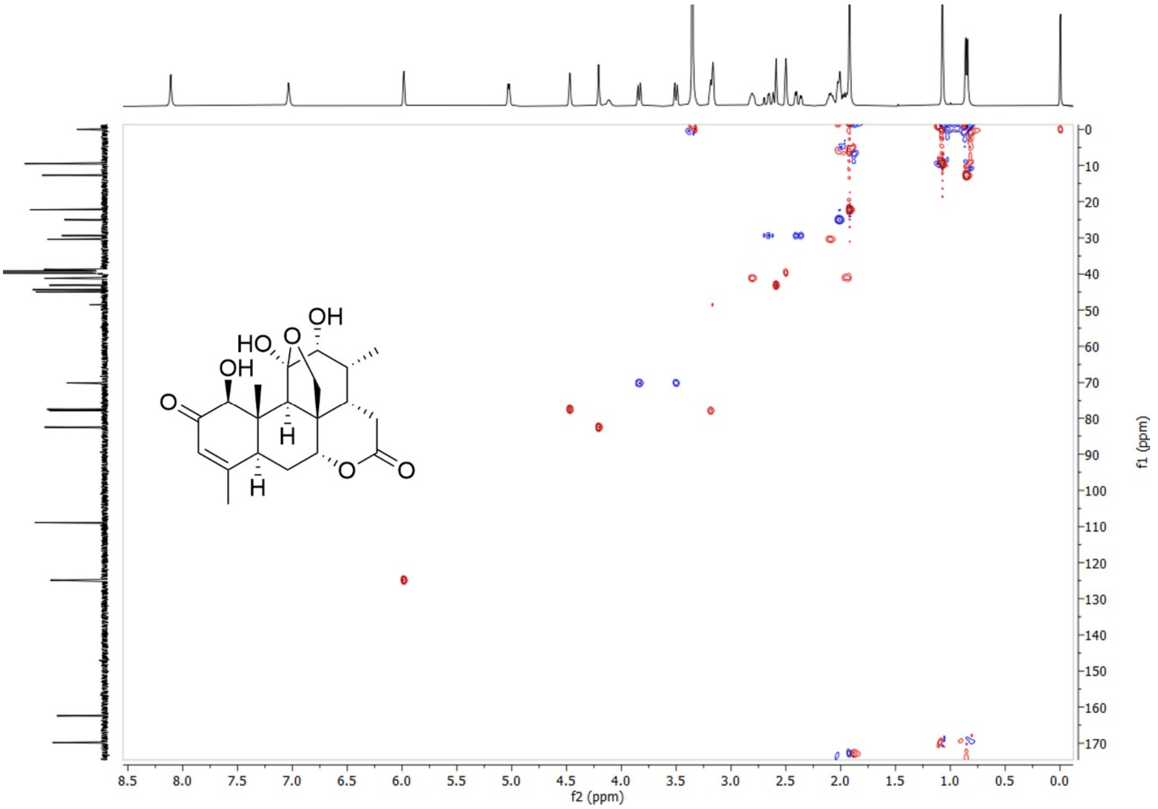

Figure S26. ^1H–^{13}C HSQC spectrum of compound **3** in DMSO-d_6 at 298 K (400 MHz).

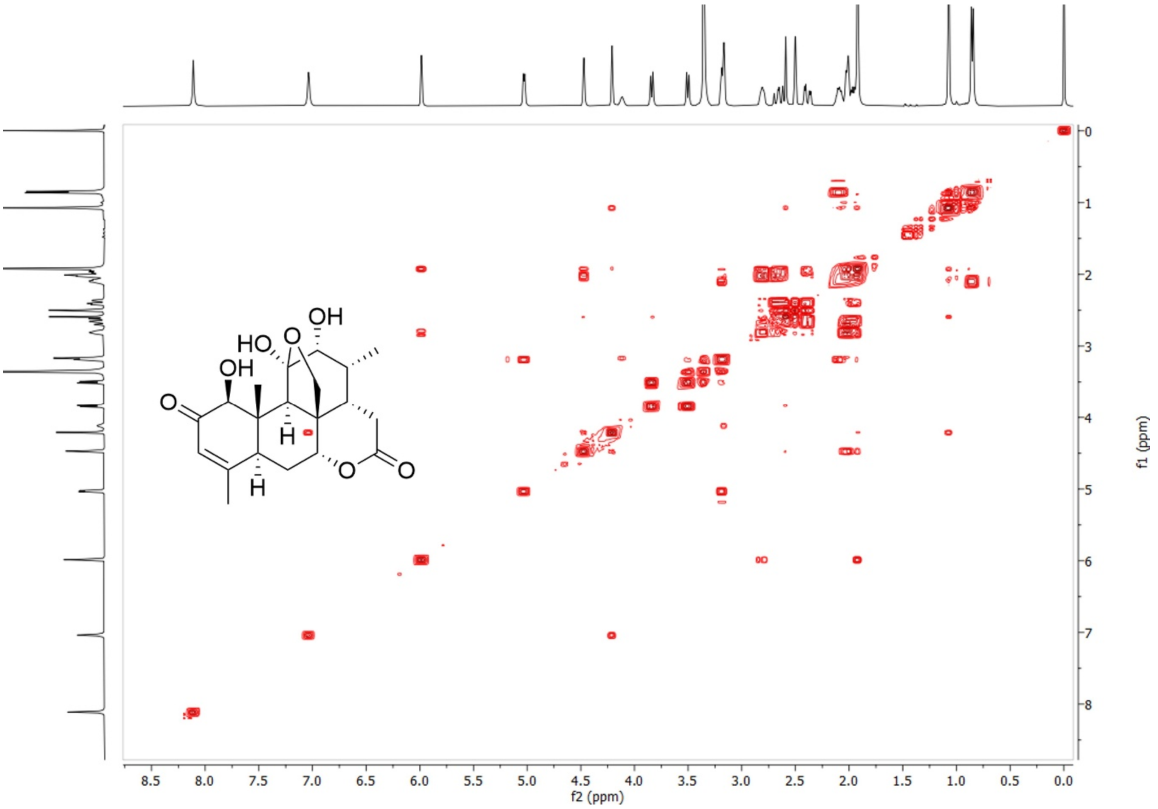

Figure S27. ^1H–^1H COSY spectrum of compound **3** in DMSO-d_6 at 298 K (400 MHz).

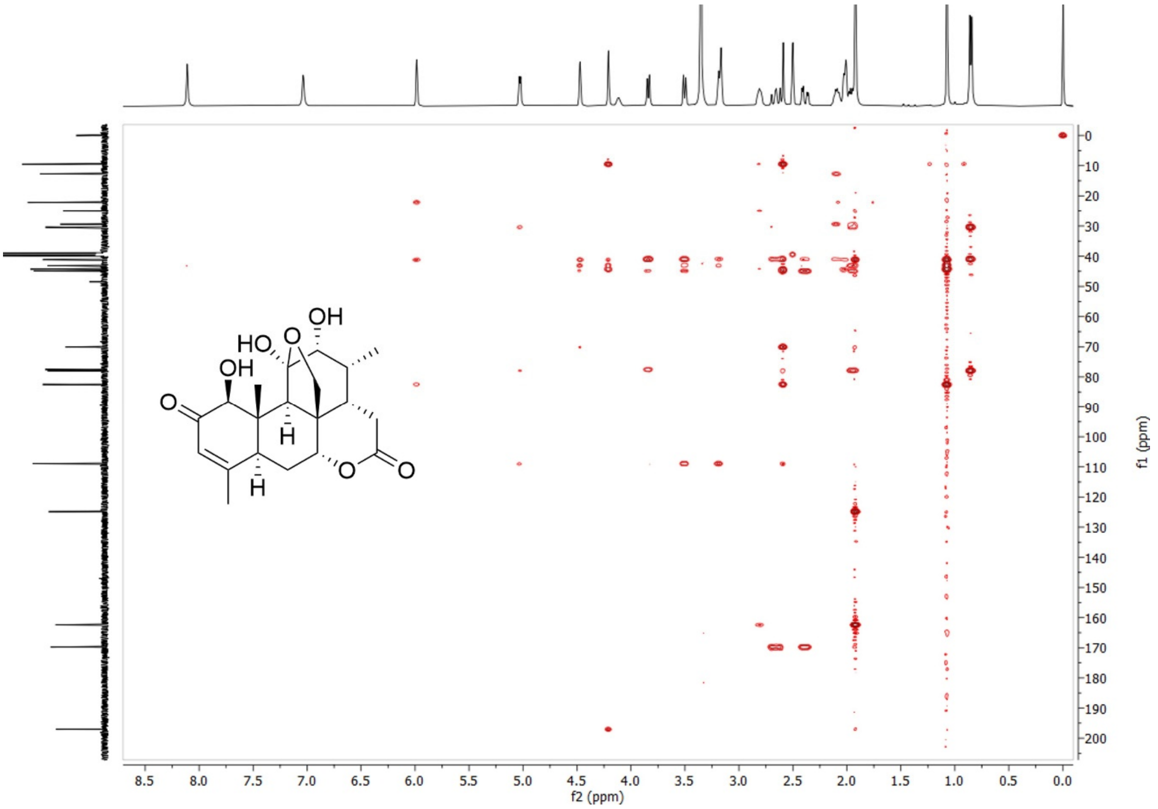

Figure S28. ^{1}H–^{13}C HMBC spectrum of compound **3** in DMSO-d_6 at 298 K (400 MHz).

Table S3. ^{1}H and ^{13}C data of compound **3**.

Position	Compound **3** (DMSO-d_6)	
	δ_C	δ_H, mult. (J in Hz)
1	82.5	4.21, s
2	197.1	-
3	124.8	5.99, s
4	162.4	-
5	41.2	2.81, m
6	25.0	2.02, d (9.2)
7	77.5	4.47, d (2.3)
8	44.9	-
9	43.1	2.59, s
10	44.3	-
11	108.9	-
12	77.9	3.18, d (8.1)
13	30.4	2.09, m
14	41.0	1.96, t (6.8)
15	29.4	2.39, dd
		2.66, dd
16	169.8	-
17	22.2	1.92, s
18	12.7	0.85, d (7.0)
19	9.5	1.07, s
20	70.1	3.50, d (8.4)
		3.84, d (8.4)
1-OH		7.04, s
11-OH		8.11, s
12-OH		5.03, d (4.5)

Table S4. Physicohemical, pharmacokinetic, and toxicologic predictive values of quassinoids and metronidazole[a].

	Chaparrin	Amarolide	Chaparrinone	Metronidazole
		Physicochemical		
MW (g/mol)	380.44	364.44	378.42	171.15
TPSA (Å^2)	116.45	100.90	113.29	83.87
LogP	1.52	1.767	1.360	−0.102
Rb	0	0	0	3
nHD	4	2	3	1
nHA	7	6	7	6
		Druglikeness		
Lipinski	Yes	Yes	Yes	Yes
Ghose	Yes	Yes	Yes	Yes
Veber	Yes	Yes	Yes	Yes
Egan	Yes	Yes	Yes	Yes
		Toxicity [b]		
Mutagenic	Inactive	Active	Inactive	Active
Carcinogenic	Inactive	Inactive	Inactive	Active
Neurotoxicity	Inactive	Inactive	Inactive	Inactive
Nephrotoxicity	Inactive	Inactive	Inactive	Active
H-HT	+	--	-	+++
Cardiotoxicity	Inactive	Inactive	Inactive	Inactive
ROAT	++	+	++	---
Predicted Toxicity Class [b]	4	5	2	4

	Chaparrin	Amarolide	Chaparrinone	Metronidazole
		Pharmacokicetic		
		Absorption		
HIA (%)	67.73	70.89	70.3	80.28
Caco-2 (log cm/s)	-5.730	-5.076	-5.448	-4.712
		Distribution		
PPB (%)	41.961	42.807	34.831	17.40
BBB (%)	25.53	15.07	25.74	32.36
VD (L/kg)	0.657	0.515	1.230	0.830
		Metabolism		
CYP1A2 inhibitor	---	---	---	---
CYP2C19 inhibitor	---	---	---	---
CYP2C19 substrate	-	+	+	+
CYP2C9 inhibitor	---	---	---	---
CYP2D6 inhibitor	---	---	---	---
CYP2D6 substrate	--	--	--	-
CYP3A4 inhibitor	---	--	---	---
		Excretion		
Clearance (mL/min/kg)	2.607	13.081	2.665	6.299
T1/2 (h)	0.825	0.826	0.796	0.525

[a] Predictions were based on SwissADME, ADMET*lab*, ai-druglab and Tox-prediction web servers. For the classification endpoints, the prediction probability values are transformed into six symbols: 0-0.1 (---), 0.1-0.3 (--), 0.3-0.5 (-), 0.5-0.7 (+), 0.7-0.9 (++), and 0.9-1.0 (+++). Molecular weight (MW), logarithm of the n-octanol/water distribution coefficient (LogP), water solubility (LogS), topological polar surface area (TPSA), number of H-bond acceptors (nHA), rotatable bonds (Rb), human intestinal absorption (HIA), plasma protein binding (PPB), blood–brain barrier permeability (BBB), volume of distribution (VD), half-life (T1/2), Human hepatotoxicity (H-HT) and Rat Oral Acute Toxicity (ROAT). [b] Class I: fatal if swallowed ($LD_{50} \leq 5$), Class II: fatal if swallowed ($5 < LD_{50} \leq 50$), Class III: toxic if swallowed ($50 < LD_{50} \leq 300$), Class IV: harmful if swallowed ($300 < LD_{50} \leq 2000$), Class V: may be harmful if ingested ($2000 < LD_{50} \leq 5000$), Class VI: non-toxic ($LD_{50} > 5000$).
